# Differential diagnosis of iron deficiency anemia from aplastic anemia using machine learning and explainable Artificial Intelligence utilizing blood attributes

**DOI:** 10.1038/s41598-024-84120-w

**Published:** 2025-01-02

**Authors:** B. S. Dhruva Darshan, Niranjana Sampathila, G. Muralidhar Bairy, Srikanth Prabhu, Sushma Belurkar, Krishnaraj Chadaga, S. Nandish

**Affiliations:** 1 Department of Biomedical Engineering, Manipal Institute of Technology, Manipal Academy of Higher Education, Manipal, Karnataka 576104 India; 2 Department of Computer Science & Engineering, Manipal Institute of Technology, Manipal Academy of Higher Education, Manipal, Karnataka 576104 India; 3 Hematology and Clinical Pathology lab, Kasturba Medical College, Manipal Academy of Higher Education, Manipal, Karnataka 576104 India; 4 L&T Technology Services Limited, Mysore, Karnataka India

**Keywords:** Explainable Artificial Intelligence, Iron deficiency anemia, Aplastic Anemia, Machine learning, Classification, Ensemble models, Biomarkers, Diseases, Health care

## Abstract

As per world health organization, Anemia is a most prevalent blood disorder all over the world. Reduced number of Red Blood Cells or decrease in the number of healthy red blood cells is considered as Anemia. This condition also leads to the decrease in the oxygen carrying capacity of the blood. The main goal of this research is to develop a dependable method for diagnosing Aplastic Anemia and Iron Deficiency Anemia by examining the blood test attributes. As of today, there are no studies which use Interpretable Artificial Intelligence to perform the above differential diagnosis. The dataset used in this study is collected from Kasturba Medical College, Manipal. The dataset consisted of various blood test attributes such as Red Blood cell count, Hemoglobin level, Mean Corpuscular Volume, etc. One of the trending topics in Machine Learning is Explainable Artificial Intelligence. They are known to demystify the machine learning outputs to all its stakeholders. Hence, Five XAI tools including SHAP, LIME, Eli5, Qlattice and Anchor are used to understand the model’s predictions. The importance characteristics according to XAI models are PLT, PCT, MCV, PDW, HGB, ABS LYMP, WBC, MCH, and MCHC. are employed to train and test the data. The goal of using data analytic techniques is to give medical professionals a useful tool that improves decision-making, enhances resource management, and eventually raises the standard of patient care. By considering the unique qualities of each patient, medical professionals who must rely on AI-assisted diagnosis and treatment suggestions, XAI offers arguments to strengthen their faith in the model outcomes.

## Introduction

As per the update in January 2022, the prevalence of anaemia varies globally and is influenced by factors such as age, sex, socioeconomic status, and geographical location. Anaemia is a condition characterized by a lower-than-normal level of red blood cells (RBC) or haemoglobin (HGB) in the blood, leading to a reduced capacity of the blood to carry oxygen to body tissues^1^. The World Health Organization (WHO) estimated that approximately 1.62 billion people worldwide were affected by anemia, representing nearly 25% of the global population^2^. However, it’s important to note that these estimates may have changed since then, and the prevalence of anemia can be subject to fluctuations over time. HGB is a protein in RBC that carries oxygen from the lungs to the rest of the body and returns carbon dioxide from the body to the lungs^3^. Anemia occurs when there is a deficiency of RBC or HGB, leading to a reduced ability of the blood to carry oxygen. HGB levels are a key indicator in the diagnosis and classification of anemia. The WHO defines anemia based on HGB levels, and the thresholds may vary by age, sex, and pregnancy status^4^.

Given below the general HGB concentration thresholds used to define anemia in adults:

1. For adult males: HGB levels below 13 g per decilitre (g/dL) may indicate anemia.
2. For adult non-pregnant females: HGB levels below 12 g/dL may indicate anemia.
3. For pregnant females: HGB levels below 11 g/dL may indicate anemia.

**Fig. 1:**
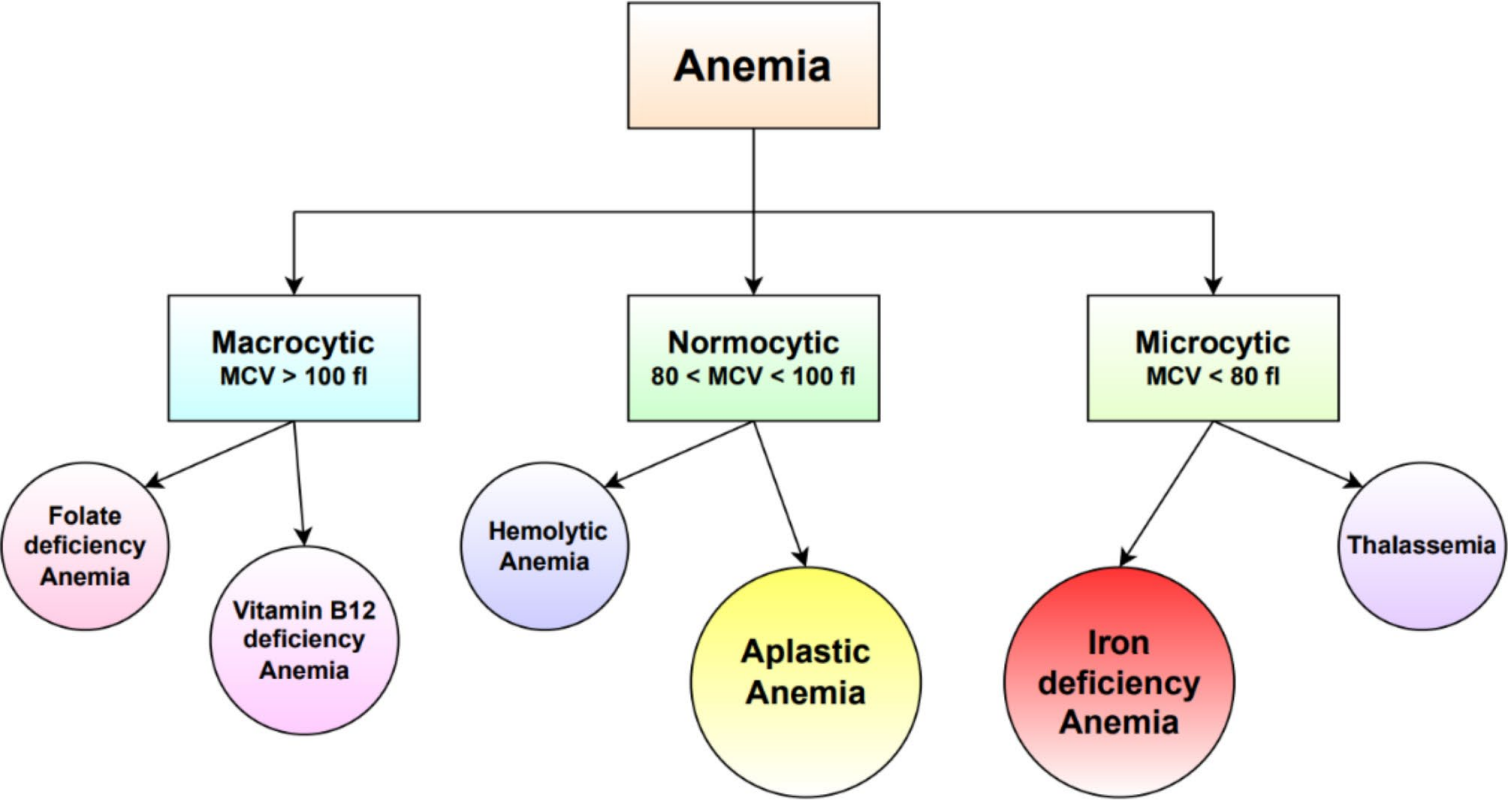
Classification of anemia based on mean corpuscular volume.

There are several types of anemia, each with its own causes and characteristics. Some common types of anemia are Iron deficiency anaemia (IDA), Vitamin B12 deficiency anemia, Aplastic Anaemia (AA), Folate deficiency anemia, Sickle cell anemia etc^5^. Figure 1 shows the classification of anaemia based on Mean Corpuscular Volume (MCV). If MCV is lesser than 80 Femtolitre (fL) the anaemia is called Microcytic anaemia. If MCV is in the range 80–100 fL then it is Normocytic anaemia. And if the MCV is greater than 100 fL then it is Macrocytic anaemia^6^. IDA falls under Microcytic anaemia and AA falls under the Normocytic anaemia.

The severity and specific symptoms can vary depending on the underlying cause, the degree of anemia, and individual factors. The effects of anemia can vary based on the underlying cause. Common causes include iron deficiency, vitamin B12 deficiency, folic acid deficiency, chronic diseases, and genetic conditions^7^. It’s important to consult with a healthcare professional for proper diagnosis and treatment if anemia is suspected. Treatment may involve addressing the underlying cause, nutritional supplements, dietary changes, or other medical interventions^8^.

Machine learning (ML) plays a significant role in revolutionizing various aspects of healthcare, offering opportunities for improved diagnostics, treatment planning, personalized medicine, and administrative processes^9^. Despite the potential benefits, the implementation of ML in healthcare also raises challenges related to data privacy, security, interpretability, and ethical considerations. Striking a balance between technological innovation and ethical practices is crucial for the responsible deployment of ML in the healthcare sector^10^.

“XAI” in healthcare stands for Explainable Artificial Intelligence. Explainable AI refers to the development of AI systems in a way that allows humans to understand how these systems arrive at their decisions or predictions^11^. This transparency is crucial, especially in sectors like healthcare, where trust, accountability, and the ability to interpret AI outputs are paramount. Efforts are ongoing in the research and development of XAI techniques to make AI models in healthcare more transparent and interpretable. Striking a balance between the complexity of AI algorithms and the need for transparency is crucial for the successful integration of AI into healthcare settings^12^.

In this study, we focus on the differential diagnosis of Iron deficiency anemia from aplastic anemia. This is a challenging task since there are a huge number of over lapping symptoms. Even laboratory findings are very similar to each other. Hence, several machine learning algorithms and XAI methodologies have been developed to solve this use-case^2–4^.

Several studies have already employed ML and Deep Learning (DL) algorithms for the diagnosis of anemia. The research efforts listed below have substantially increases our understandings. Kilicarslan et al. in 2020^13^ developed a hybrid model for the anemia classification. It proposes two hybrid models, GA-SAE, and GA-CNN, integrating genetic algorithms with Stacked Autoencoder (SAE) and Convolutional Neural Network (CNN) to predict various anemic types and non-anemic conditions. Model is evaluated with Accuracy of 98.50%. This suggests the effectiveness of the hybrid approach in addressing the complexities of anemia prediction and classification. Zhang et al. in^14^ proposed a DL system to predict anemia in emergency department patients based on facial characteristics, aiding in rapid diagnosis and treatment decisions, particularly regarding blood transfusions. The study achieved promising accuracy and sensitivity levels across different degrees of anemia. The work demonstrates the system’s potential for clinical use in expediting diagnosis and resource allocation. This work aligns with prior research emphasizing the importance of accurate predictive models in healthcare, particularly in emergency settings and underscores the clinical value of ML technology in improving patient care. Appiahene et al. in^15^ demonstrated the application of ensemble models approach in anemia detection. They utilized images of the palpable palm to develop ML models for anemia detection. It highlights the importance of early detection, especially in resource-limited settings where non-invasive methods are preferred. Ensemble learning techniques including stacking, voting, boosting, and bagging are employed for the development build hybrid models. Stacking ensemble model achieved high accuracy of 99.73%. The study underscores the efficiency of ensemble models in medical diagnosis, particularly for diseases like anemia and suggests their potential to improve healthcare accessibility and affordability. Zaimoku et al.^16^ examined the efficacy of combining eltrombopag with standard immunosuppressive therapy for severe aplastic anemia treatment (SAA). It investigates predictors of treatment response in 416 SAA patients, finding that pretreatment blood counts, especially absolute reticulocyte counts, correlate with response. The addition of eltrombopag improves overall response rates, particularly in patients with lower reticulocyte counts. However, platelet count and the presence of paroxysmal nocturnal haemoglobinuria clones did not correlate with treatment responses. The study underscores the significance of blood counts in predicting response to non-transplant therapies in SAA. El-Kenawy et al. in^17^ demonstrated the estimation of anemia for COVID-19 patients using a ML model. The work gives the importance of hemoglobin levels. HGB evaluation model preprocesses the data for training and achieves highly accurate results compared to other ML models. This approach offers valuable insights for physicians in understanding CBC changes in COVID-19 patients, potentially improving diagnostic and monitoring processes.

Aliyu et al. in^17^ presented a DL AlexNet model for the classification of RBC in Sickle Cell Anemia (SCA) using blood smear images. The proposed model involves two phases: automating RBC extraction and employing DL AlexNet model for classification. Using over 9,000 RBC images from 130 SCA patients, the framework achieves automated classification of 15 RBC shapes, including normal. The study achieved high accuracy of 95.92%. This approach offers a promising solution for improving SCA diagnosis and management, reducing reliance on manual microscopy and expert interpretation. Kinyoki et al. in^18^ studied the prevalence of anemia in women of reproductive age in low- and middle-income countries between 2000 and 2018. It assesses the progress towards the WHO Global Nutrition Target (GNT) to reduce anemia by 2030. While moderate improvements are noted overall, only three countries are expected to meet the target nationally, with disparities within countries remaining significant. The findings highlight the need for targeted interventions to address anemia among vulnerable populations, guiding resource allocation and public health efforts.

Dejene et al. in^19^ focused on predicting anemia levels among pregnant women in Ethiopia using ML algorithms. Using data from the Ethiopian Demographic Health Survey, the study employs DT, RF catboost, and extreme gradient boosting algorithms. Catboost achieved highest accuracy of 97.6%. Key risk factors for anemia include pregnancy duration, age, water source, occupation, household size, wealth index, partner’s education, and birth history. This information can guide targeted interventions to improve maternal and prenatal health outcomes in Ethiopia. Appiahene et al. in^20^ did a comparative study of ML algorithms in the detection of IDA by medical images. The study focuses on non-invasively detecting anemia using palm images and ML algorithms. Among various models used, NB achieved highest accuracy of 99.96%. This highlights the efficiency and effectiveness of ML in detecting anemia, supporting its potential as a non-invasive diagnostic tool for IDA.

Shwetha et al. in^21^ examined the prediction of anemia using various Ensemble learning and Boosting techniques. Various ML algorithms including RF, SVM, NB, Linear discriminant analysis (LDA), Quadratic Discriminant Analysis (QDA), and ensemble methods are evaluated for accuracy and execution time. XGB emerges as the best-performing model, providing optimal accuracy within a reasonable execution time. This highlights the effectiveness of ML in predicting anemia and underscores XGB as a preferred model for this task.

The literature survey highlights the diverse application of ML in anemia classification, showcasing its potential for accurate diagnosis and personalized treatment recommendations. However, challenges such as data quality and interpretability remain, emphasizing the need for further collaboration between ML experts and medical practitioners.

While our research has made significant strides in the realm of anemia diagnosis through the integration of ML and XAI, certain gaps in the existing literature merit attention. First and foremost, there remains a dearth of comprehensive studies that explicitly address the interpretability of anemia prediction models in the context of diverse populations and healthcare settings. Moreover, the current body of literature lacks a unified approach to handling ethical considerations, data privacy concerns, and bias mitigation strategies specific to anemia diagnosis using advanced AI techniques. The literature also falls short in providing a nuanced understanding of the impact of Explainable AI on the decision-making process of healthcare practitioners, necessitating further exploration into the acceptance and integration of such technologies into clinical workflows.

The goal of the current work is to improve anemia prediction by utilizing XAI techniques such as SHAP, LIME, Qlattice, Eli5 and Anchor. XAI, a new advancement in ML, seeks to answer the open question of how “black box” AI algorithms arrive at judgements. This field studies decision-making processes and models in an attempt to improve their comprehension and comprehensibility. A detailed and comprehensive summary of the relevant work is given in Table 1. As of today, there are no articles which differentiate iron deficiency anemia from aplastic anemia.

**Table 1:** Overview of the related work.

Reference	Dataset used	ML algorithms	Results	Pros	Cons
Zemarium et al.^22^	5642 weighted samples from the 2016 Ethiopian Demographic and Health Survey dataset.	SVM, GNB, LR, DT, RF, LGB, XGB, KNN	RF achieved the highest performance with an AUC value of 82%.	Identification of key predictors for anemia among young girls. Emphasis on the potential utility of predictive models for decision-making and intervention planning.	Reliance on a single dataset may limit generalizability. Lack of validation on independent datasets. Potential bias or inaccuracies in self- reported survey data. Limited discussion on the interpretability of ML models and practical implementation considerations.
Rahman et al.^23^	1000 instances and 8 attributes sourced from a local pathology center.	LR, NB, KNN, DT, SVM, RF, ADB, SGB, XGB, Ridge and Bagging classifier.	LR achieved the highest accuracy of 95% in predicting anemia.	Focus on early detection of anemia using ML techniques. Utilization of ensemble learning for improved prediction performance.	Limited description of dataset characteristics and preprocessing techniques. Lack of discussion on potential limitations or biases in the dataset. Absence of external validation on independent datasets.
Qasravi et al.^24^	Balanced dataset of 755 female participants.	K-means clustering and Decision tree algorithms.	Found 34.8% of participants were anemic. Decision tree achieved accuracy of 82.1%.	Identification of novel nutrients associated with anemia in university students. Practical implications for nutrition intervention and anemia prevention.	Limited discussion on potential confounding factors or biases in data collection. Generalizability may be limited to university students from specific demographic regions. Absence of discussion on interpretability of Decision Tree models.
Siddartha et al.^25^	US NHANES dataset of over 19,000 instances. Unseen dataset from Kenya for validation.	LR, RF, KNN, Gradient boosting, XGB	Gradient boosting classifier achieved highest accuracy of 87%.	Cost-effective and widely available diagnostic approach using CBC data. Consistency of results across different datasets and robustness of explanations.	Limited discussion on potential biases or limitations in the datasets. Absence of comparison with existing diagnostic methods. Lack of discussion on interpretability of ML models for healthcare professionals.
Asare et al.^26^	Local dataset.	KNN, NB, DT, SVM, CNN	CNN achieved highest accuracy of 98.45%.	Utilization of ML algorithms for non-invasive detection of anemia. Emphasis on cost-effectiveness and sustainability for resource constrained communities.	Absence of external validation on independent datasets. Need for further exploration of scalability and real-world implementation challenges.
Saputra et al.^27^	Retrieved historical data from the clinical pathology lab, Universitas Gadjah Mada, Indonesia.	Extreme Learning Machine algorithm	Achieved accuracy of 99.21%.	Using ML algorithm for automated prediction of anemia types. Potential to improve diagnosis process and facilitate personalized treatment.	Need for further exploration of scalability and real-world implementation challenges. Limited discussion on potential biases or limitations in the dataset.
Kassaw et al.^28^	Cross-sectional study design with Ethiopian Demographic and Health Survey 2016 dataset.	Data analysis using statistical Package for Social sciences (SPSS), R-software and Boruta algorithm	Identified top predictors for anemia among under-five children.	Identification of key predictors informs policy and intervention strategies for anemia prevention and control. Integration of Boruta algorithm for feature selection enhances model interpretability.	Reliance on secondary data source may introduce biases or limitations. Absence of external validation on independent datasets. Need for further exploration of casual relationships between identified predictors and anemia outcomes.
Khawaga et al.^29^	CBC test data of 8544 records downloaded from Kaggle.	RF, DT, MLP, KNN, LR, SVM	RF, MLP and DT achieved high accuracy of 99.94%.	Utilization of supervised machine learning methods for anemia prediction. Potential to improve preventive strategies and treatment plans for diseases.	Need for further exploration of scalability and real-world implementation challenges. Lack of discussion on interpretability of ML models for healthcare professionals.
Zahirzada et al.^30^	Dataset of 350 samples.	KNN, NB, MLP, RF, SVM	RF achieved highest accuracy of 86.4%.	Potential to inform health and prevention policy. Utilization of ML techniques for predictive modelling of anemia.	Limited discussion on data collection methodology and potential biases in hospital data. Absence of external validation on independent datasets. Lack of discussion on interpretability of ML models.

The findings cited above indicate that prediction has already been done using ML and AI algorithms Based on the research we are reported here the major contributions:

1. The Experiments are conducted based on our own dataset collected and prepared with 24 attributes collected from Kasturba Medical College, Manipal.
2. Feature visualization is carried out using violin plot and Pearson’s correlation and feature selection based on Mutual information is carried out for the selection of prominent attributes.
3. Basic ML models have been optimized and results of that are compared with the customized ensemble models for the differential diagnosis of IDA and AA.
4. In this unique investigation, five XAI algorithms were utilized to clarify models with the following methods: SHAP, LIME, QLattice, Eli5 and Anchor.

## Materials and methods

### Dataset description

The AA and IDA dataset having blood test attributes were obtained from the Kasturba Medical College, Manipal Academy of Higher Education. The above hospital is situated in Udupi District, Karnataka, India. Ethical clearance has been obtained to collect patient data from Manipal Academy of Higher Education ethics committee with id IEC1 :229/2022. The need for informed consent was waived by ethics committee/ Institutional Review board of Manipal Academy of Higher Education, because of the retrospective nature of the study. All methods were carried out in accordance with relevant guidelines and regulations. Patients who had anaemia during the year 2022 was taken into consideration for this research. The dataset had 500 samples, where 266 samples are of IDA and 234 samples are of AA. Thorough description of the attributes in the dataset are shown in Table 2.

**Table 2:** Attributes available in the dataset.

Attribute no.	Attribute name	Attribute type	Feature	Description
1	Age	Numerical- continuous	Clinical	Age of the individual
2	Gender	Quantitative	Clinical	Gender: 0 = male and 1 = female
3	ESR	Quantitative	Clinical	Erythrocyte sedimentation rate
4	HGB	Quantitative	Clinical	Hemoglobin
5	HCT	Quantitative	Clinical	Hematocrit
6	RBC	Quantitative	Clinical	Red blood cells
7	MCV	Quantitative	Clinical	Mean corpuscular volume
8	MCH	Quantitative	Clinical	Mean Corpuscular Hemoglobin
9	MCHC	Quantitative	Clinical	Mean Corpuscular Hemoglobin Concentration
10	RDW	Quantitative	Clinical	Red cell distribution width
11	PLT	Quantitative	Clinical	Platelets
12	MPV	Quantitative	Clinical	Mean platelet volume
13	PCT	Quantitative	Clinical	Procalcitonin
14	PDW	Quantitative	Clinical	Platelet distribution width
15	WBC	Quantitative	Clinical	White blood cells
16	NEUT	Quantitative	Clinical	Neutrophil
17	LYMP	Quantitative	Clinical	Lymphocytes
18	MONO	Quantitative	Clinical	Monocytes
19	EOSI	Quantitative	Clinical	Eosinophil
20	BASO	Quantitative	Clinical	Basophil
21	ABS NEUT	Quantitative	Clinical	Absolute neutrophil
22	ABS EOSI	Quantitative	Clinical	Absolute eosinophil
23	ABS LYMP	Quantitative	Clinical	Absolute lymphocytes
24	ANE TYPE	Quantitative	Target	Anemia class information (0 = iron deficiency anemia); (1 = aplastic anemia)

### Data preparation

Data preparation entails several procedures, including data balancing, variable encoding, data normalization, outlier removal, and null value removal. There are 23 attributes in the dataset of which some are continuous, categorical, and some are discrete values. The Mean, Median, Missing values, Standard deviation of each attribute can be found in the analysis. The missing values are replaced using mean, median and other imputation methods. Since outliers have no effect on the continuous attributes, we substituted the median for them. Gender, the categorical variable had no missing values. “Jamovi” an open- source statistical tool was used in this study to carry out a descriptive statistical analysis^31^. Several statistical metrics, including mean, median, standard deviation, interquartile range, and percentiles for some attributes are described in the Table 3.

**Table 3:** Descriptive of the dataset.

Descriptives									
							Percentiles		
Attribute	ANE TYPE	*N*	Missing	Mean	Median	SD	25th	50th	75th
PCT	0	265	1	0.2953	0.2550	0.3580	0.1960	0.2550	0.3380
	1	234	0	0.0734	0.0600	0.0650	0.0280	0.0600	0.0985
PLT	0	266	0	341.4135	314.5000	164.8522	237.0000	314.5000	413.2500
	1	234	0	85.4094	68.0000	78.3782	30.2500	68.0000	110.5000
WBC	0	266	0	10.4898	8.9000	5.9796	6.9000	8.9000	12.6000
	1	234	0	4.4800	3.1000	8.4202	1.8000	3.1000	4.9000
RBC	0	266	0	4.4356	4.2400	4.9639	3.7025	4.2400	4.6475
	1	234	0	2.7245	2.7100	0.9981	2.0650	2.7100	3.4100
ABS NEUT	0	264	2	6.2498	5.0550	4.9369	3.4675	5.0550	7.4975
	1	233	1	2.5939	1.5800	5.1416	0.8600	1.5800	2.6700
MCV	0	266	0	72.6617	71.5500	13.4597	63.9000	71.5500	80.7000
	1	234	0	91.5130	89.4500	13.3612	84.0250	89.4500	99.0000
MCH	0	266	0	24.7030	23.4000	17.4976	19.9250	23.4000	26.5750
	1	234	0	30.7833	30.2000	5.6951	27.7000	30.2000	33.5000
ABS LYMP	0	242	24	2.9172	2.1150	2.3534	1.4225	2.1150	3.6275
	1	207	27	1.1333	0.8900	1.1315	0.5850	0.8900	1.3050
PDW	0	265	1	16.5853	16.7000	8.2983	16.0000	16.7000	17.2000
	1	234	0	18.5291	17.7000	14.8281	16.9250	17.7000	18.5000
MCHC	0	266	0	31.4936	32.0000	2.4354	30.4500	32.0000	33.0000
	1	234	0	33.7321	33.8000	2.9384	32.7250	33.8000	34.7000
HGB	0	266	0	9.4098	9.6000	1.9279	8.3000	9.6000	10.7000
	1	234	0	8.0543	8.1000	2.5029	6.2000	8.1000	9.6750
HCT	0	266	0	29.7718	30.0000	5.4591	26.8000	30.0000	33.1750
	1	234	0	24.1158	24.0000	7.6369	19.4000	24.0000	29.2500

**Fig. 2:**
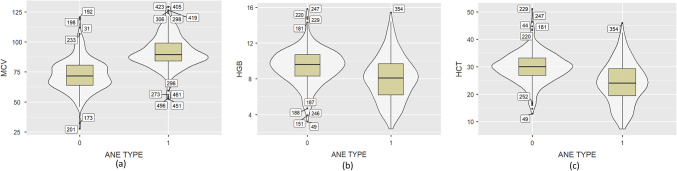
Violin plots with respect to (**a**) MCV, (**b**) HGB, (**c**)HCT.

Some of the visualization techniques are used here for finding the quartile ranges of the attributes. Box plot, Density plot, Histogram plot, Violin plots and Bar plot are some of the visualization techniques that are available. Bar graph and Violin plots were generated for the better visualization of the Dataset^32,33^. The data shown in Fig. 2 indicates the violin plot for MCV, HGB and HCT. Figure also indicates the MCV levels were slightly increased in Aplastic anemia. HGB and HCT levels are elevated in IDA. There were also outliers in some attributes. In order to prevent the models from becoming more biased during testing, we did not handle outliers in this investigation.

**Fig. 3:**
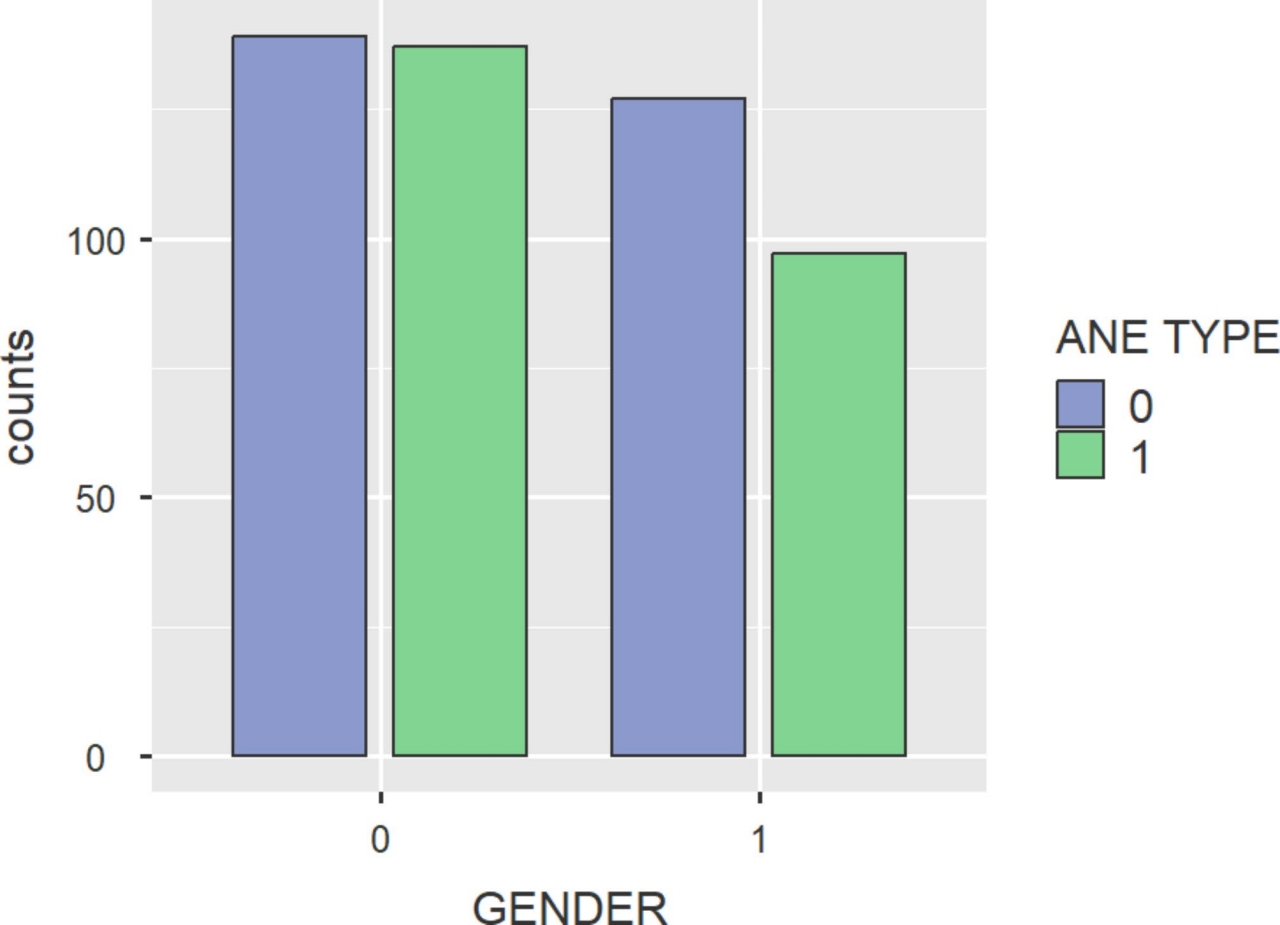
Bar plot with respect to gender count.

Bar plot gives the information about the samples available in the dataset. As mentioned earlier, the dataset used here had a total of 500 samples. Figure 3 describes the gender distribution of the dataset. There were 141 men and 125 women having IDA. And 136 men and 98 women are having AA. Total of 266 patients were having IDA and 234 patients having AA.

**Fig. 4:**
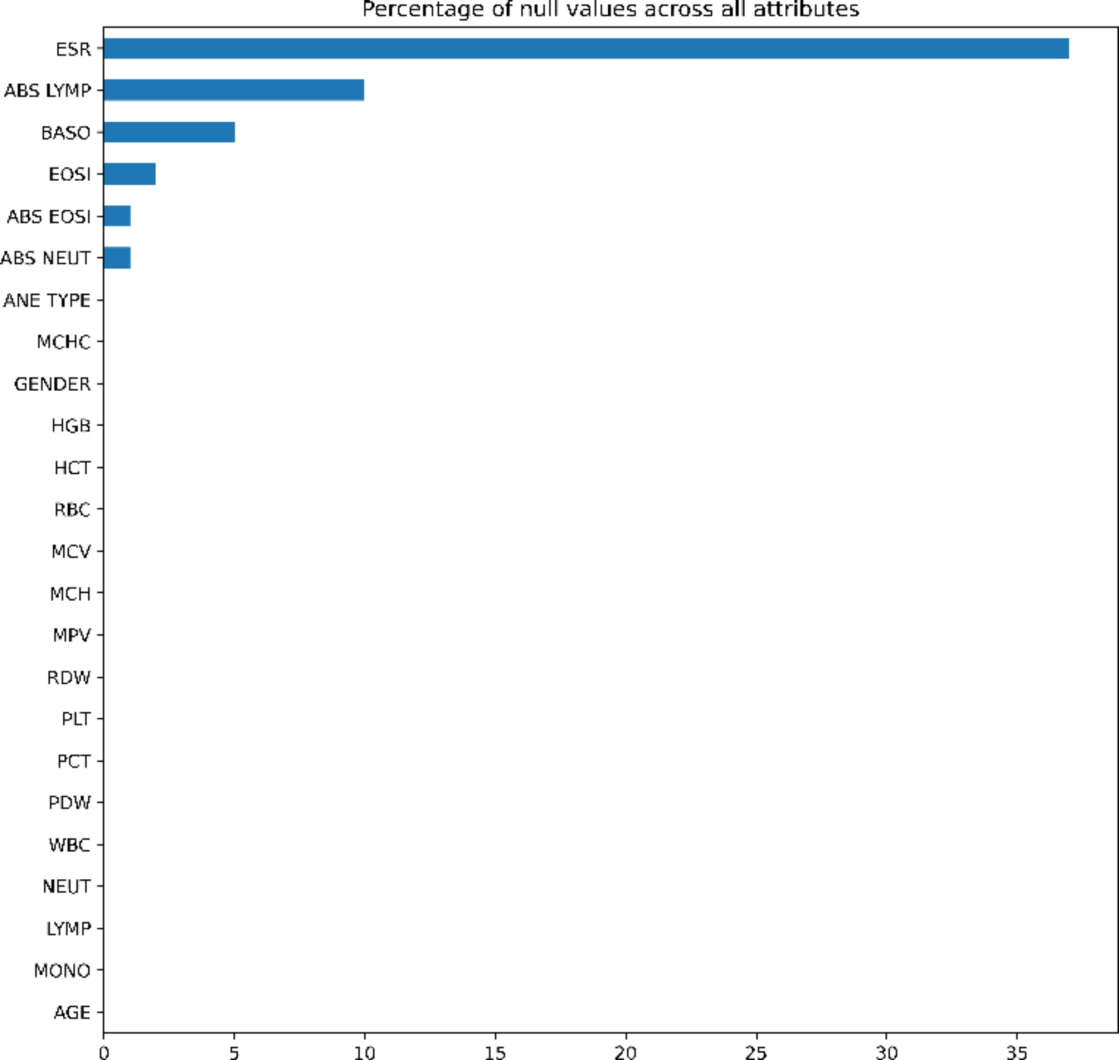
Percentage of null values in the dataset.

When there are no values present in a dataset it can be considered as null values^34^. The percentage of null values across all attributes is shown in the Fig. 4. In present dataset, ESR attribute has the most number of Null values. The other attributes which are having the null values are ABS LYMP, BASO, EOSI, ABS EOSI, ABS NEUT.

**Fig. 5:**
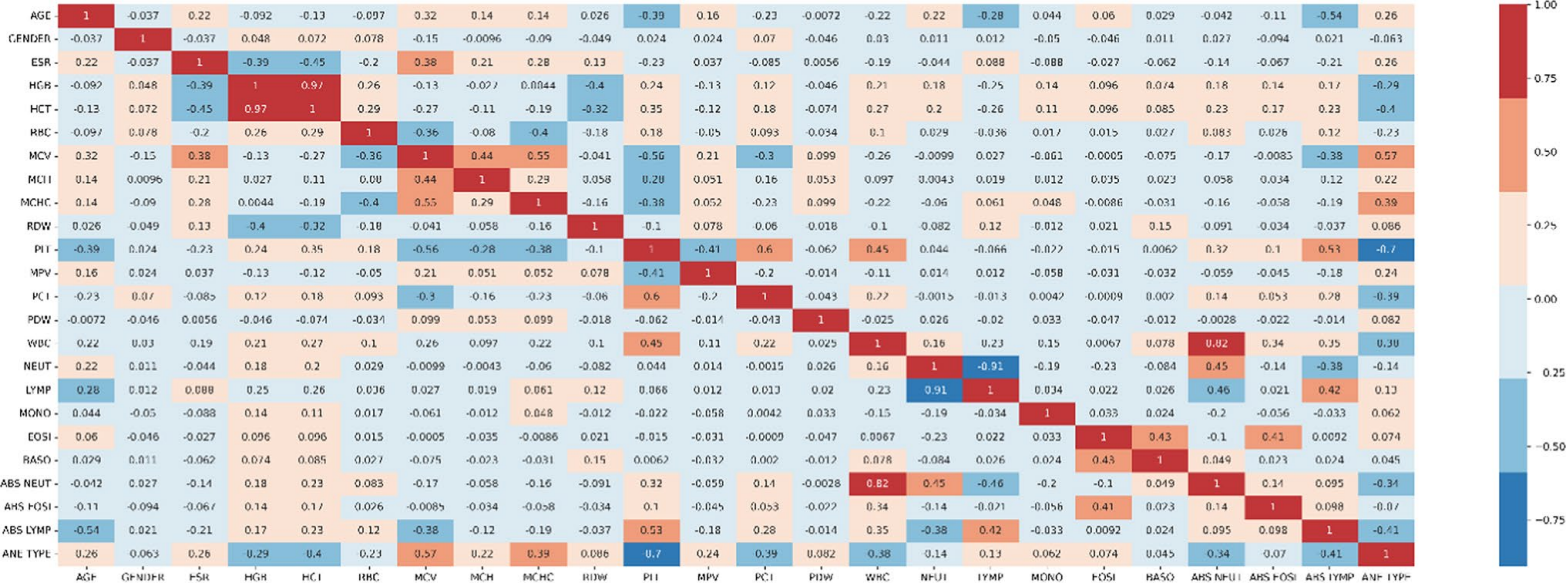
Pearson’s correlation coefficient matrix.

Some of the feature selection methods are used in this study for choosing the suitable attributes. Pearson Correlation coefficient method and Mutual information are the two feature selection techniques used. Mutual information is known to capture non-linear relationships, it is applicable to both categorical and continuous variables, does not assume data distribution and reduces the risk of overfitting. Pearson’s correlation measures the strength and direction, is sensitive to linear relationships, is resistant to outliers and helps detect multi collinearity. After the dataset was first evaluated to determine how each attribute affected the result, Pearson’s correlation coefficient analysis was carried out. If the value came near “1/1”, both the output and the coefficient value ‘r’ were perfectly related; otherwise, the value “0” indicated no association. A positive correlation coefficient value indicates that the component had a positive impact on the result. If it was unfavourable, it had the opposite effect on the outcome^35^. The rationale behind evaluating correlation coefficients is that the value of attributes of a certain variable are connected. Some variables had negative correlations, whereas few had favourable correlations. Figure 5 shows the Pearson’s correlation coefficient matrix.

**Fig. 6:**
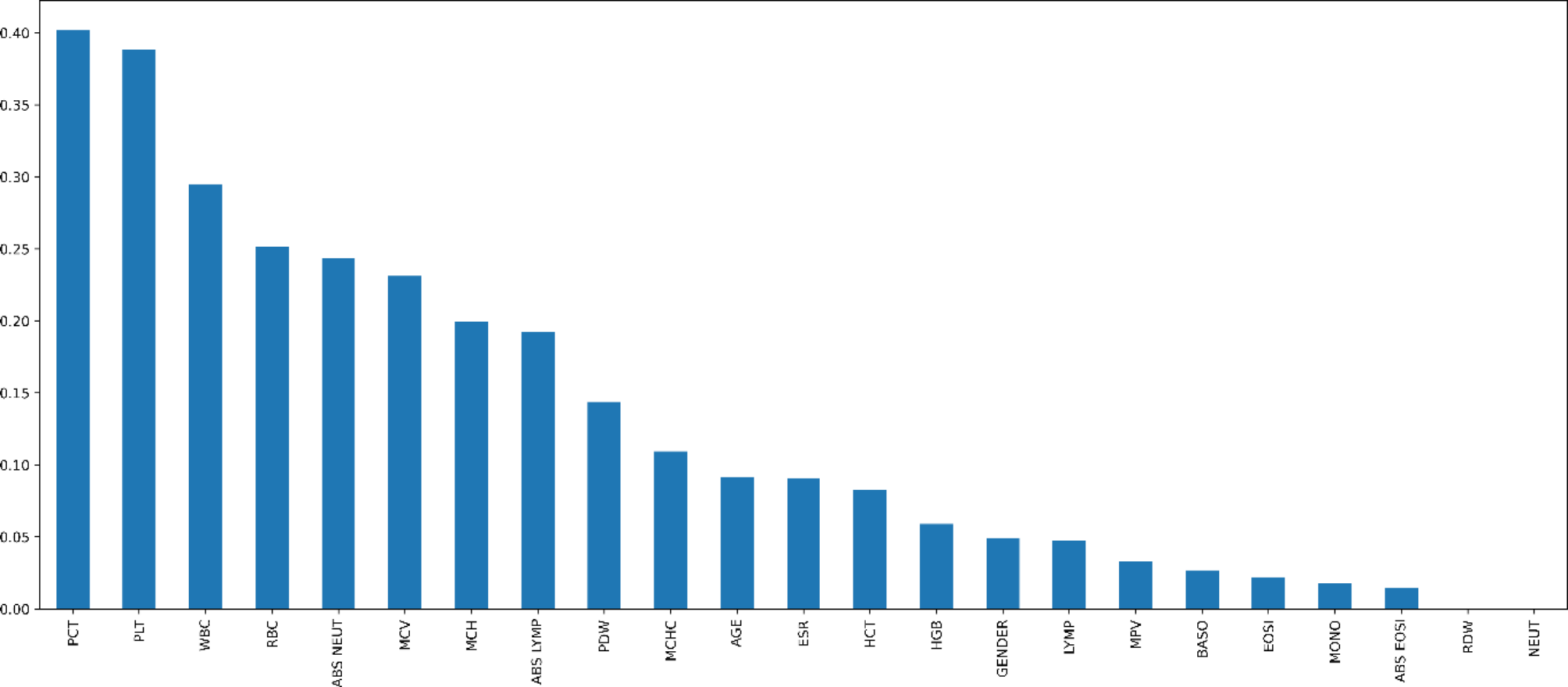
Mutual information of important attributes.

Another efficient method for choosing characteristics is the Mutual Information method. This filtering approach necessities taking the dataset’s numerical qualities into account. An entropy, or the measure of how unpredictable the features are, is necessary for mutual information^36^. As seen in Fig. 6, the attributes were ranked based on how much each contributed in relation to the desired variable.

### Machine learning

The dataset was divided into training and testing data with a ratio 80:20 after data pre-processing. The efficacy of the models is adversely affected when there is a significant disparity among the data points. Moreover, independent of the measurements used, the algorithms favor qualities with higher values. Standardization was utilized in this study to scale the data. The data points are clustered around the mean of the features during standardization, and the standard deviation of the feature is assigned as one^37^.

Medical data often exhibit imbalances, leading to distortions in the proportion of the data. Table 4 shows the descriptives of some of the attributes in the dataset. It can be seen that IDA cases are slightly higher than that of AA. As the category having more occurrences is preferred by the classifiers, Data balancing becomes crucial. In order to balance the training data, Borderline SMOTE approach is used in this study. To preserve the integrity of the data, balancing was not applied to the testing data^38^.

The model was trained using some ML algorithms. The grid search method was used to determine the optimal hyperparameters for each model^39^. The hyperparameters used for the models is shown in the Table 4. Moreover, throughout training, a fivefold cross-validation technique was used. It divides the data for testing and training into a number of subgroups. When the data are separated into folds, the models become more dependable^40^. In addition, every model was piled at different heights.

**Table 4:** Hyperparameters used.

Sl. no	Classifier	Hyperparameters
1	Logistic regression	{‘C’: 10, ‘penalty’: ‘l2’}
2	Random forest	{‘bootstrap’: True, ‘max_depth’: 100, ‘max_features’: 2, ‘min_samples_leaf’: 4, ‘min_samples_split’: 10, ‘n_estimators’: 100}
3	Decision tree	{‘criterion’: ‘entropy’, ‘max_depth’: 30, ‘max_features’: ‘auto’, ‘min_samples_leaf’: 11,‘min_samples_split’: 30, ‘splitter’: ‘best’}
4	K-nearest neighbour	{‘n_neighbors’: 15}
5	Adaboost	{‘learning_rate’: 0.001, ‘n_estimators’: 1000}
6	Cat boost	{‘border_count’: 32, ‘depth’: 3, ‘iterations’: 250, ‘l2_leaf_reg’: 1, ‘learning_rate’: 0.03}
7	Light GBM	{‘lambda_l1’: 0, ‘lambda_l2’: 1, ‘min_data_in_leaf’: 50, ‘num_leaves’: 31, ‘reg_alpha’: 0.1}
8	Xg boost	{‘colsample_bytree’: 0.3, ‘gamma’: 0.1, ‘learning_rate’: 0.15, ‘max_depth’: 3, ‘min_child_weight’: 7}
9	Stack	(classifiers=[clf1, clf2, clf3, clf4,clf5,clf6,clf7,clf8], use_probas = True, average_probas = False, meta_classifier = meta_clf)
10	ANN	(optimizer=’adam’, loss=’binary_crossentropy’, metrics=[‘accuracy’])

**Fig. 7:**
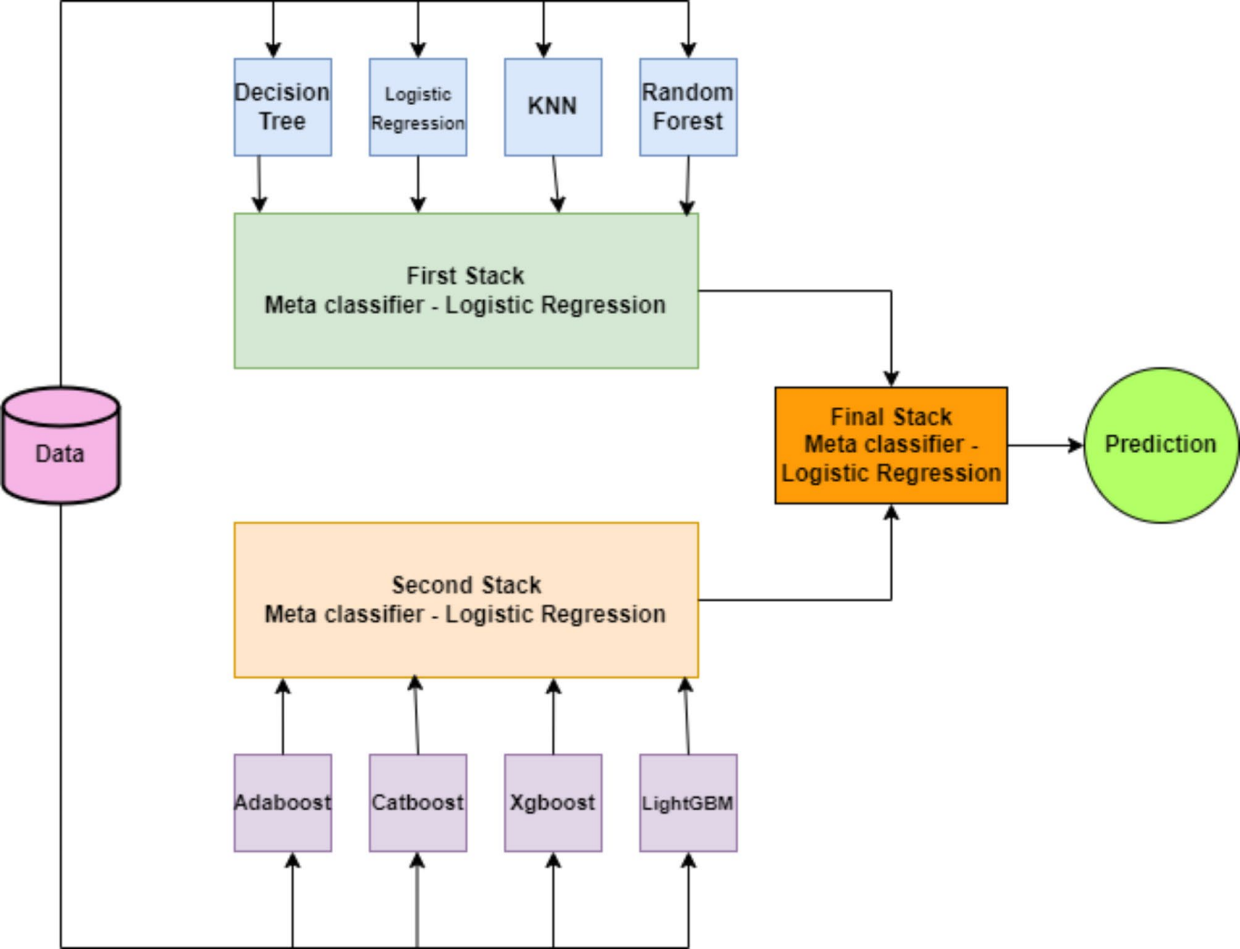
Stacking architecture.

Stacking is a collective learning technique that uses a meta-learner to aggregate the output of many classifiers^41^. The meta-learners minimize the shortcomings of the corresponding baseline classifiers while optimizing the capabilities of each model. This unique stacking method produces a classifier that is superior and more dependable. Using Logistic Regression (LR), Decision trees (DT), Random Forest (RF), and K-nearest neighbor (KNN), the first stacked model was created. To create the second stack, boosting classifiers such as Adaptive boosting (Adaboost), Extreme gradient boosting (Xgboost), Light gradient boosting machine (LGBM), and Categorical boosting (Catboost) were combined^42^. The first and second stacks were used to ensemble the final stack. Because the final stacked model incorporates multiple heterogeneous classifiers, it can be utilized for prediction. Logistic Regression was the meta-classifier employed in all stacking models. A visual representation of the modified stacking architecture can be seen in the Fig. 7.

Another component of ML is DL. DL analyses datasets using a predefined mathematical framework to find patterns and relationships. DL does the feature mining and modelling tasks automatically during training, while ML requires feature selection. In addition, it has the ability to handle unstructured data, generate new features, carry out self-learning activities, and facilitate parallel processing. Artificial Neural Networks (ANN) are computer processing systems that significantly mimic the functioning of the human brain. The basic unit of ANN is a vast network of interconnected computing nodes, or neurons. Together, these dispersed neurons maximize the result and absorb the information. The data is loaded as a multidimensional vector and then sent to the hidden layers after being received by the input layer. The hidden layers carryout the learning process by evaluating whether a stochastic alteration will ultimately improve or degrade the output based on the judgements made by the layer before it^43^.

**Fig. 8:**
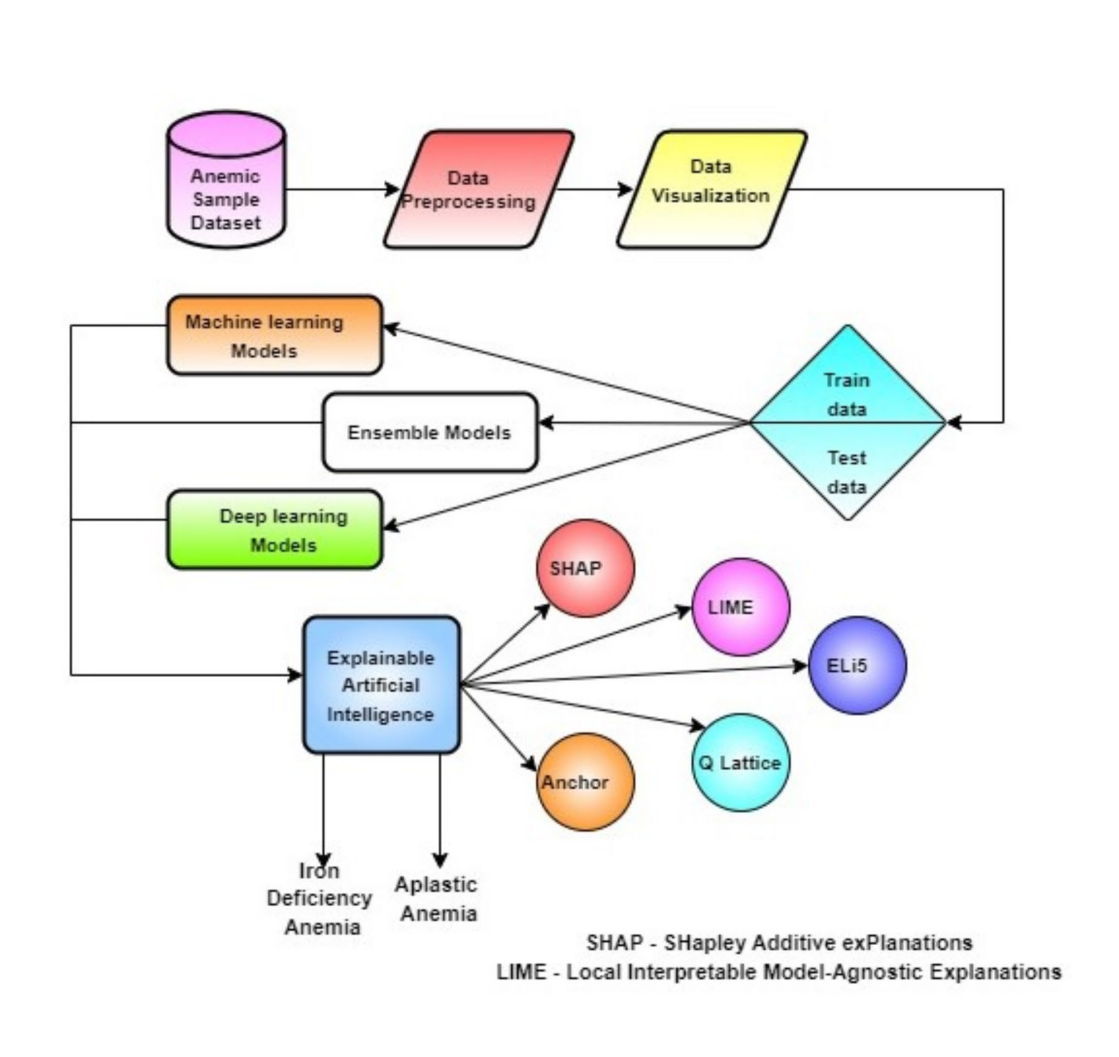
Pipelining of the IDA and AA diagnosis.

Models are intended to be understood and analyzed using a set of frameworks and tools known as XAI. In this research, the explainers used are Shapley additive values (SHAP), Local interpretable model agnostic explanations (LIME), Eli5, Qlattice, and Anchor^44^. Figure 8 shows the full flow diagram for the ML pipeline. The performance measurements that were utilized to verify the classifiers are listed in Table 5^45^.

**Table 5:** Performance measurements.

Sl. no.	Metric name	Formula	Description
1	Confusion matrix		It is a matrix used to determine the performance of the classification models for a given set of test data.
2	Accuracy	$\:\text{Accuracy=}\frac{\text{TP+TN}}{\text{TP+TN+FP+FN}}$ (1)	It measures how many correct predictions a ML model makes.
3	Precision	$\:\text{Precision=}\frac{\text{TP}}{\text{TP+FN}}$ (2)	It measures the number of positive classifications, which were correct. The precision is high when false positive results are low.
4	Recall	$\:Recall=\frac{TP}{TP+FN}$ (3)	It gauges how well the model can identify the positive class. The recall is high when false negative results are low.
5	F1-score	$\:\text{F}1-\text{s}\text{c}\text{o}\text{r}\text{e}=\frac{2\times\:Precision\times\:Recall}{Precision+Recall}$ (4)	It is a metric which combines both precision and recall.
6	AUC		In ROC curve, true positive rate is plotted against false positive rate at various thresholds. The area under the curve is called AUC (Area under curve)
7	Jaccard score (JS)	${\text{Jaccard}}\;{\text{score}} = {\text{J}}\left( {{\text{A}},{\text{B}}} \right) = \frac{{|{\text{A}} \cap {\text{B}}|}}{{|{\text{A}} \cup {\text{B}}|}}$ (5) Where, A and B are the two classes	The degree of similarity between two groups of data is gauged by the Jaccard score.
8	Log loss (LL)	${\text{Logloss}} =$ $- \frac{1}{{\text{N}}}\mathop \sum \limits_{{{\text{i}} - 1}}^{{\text{N}}} {\text{y}}_{i} \times \log \left( {{\text{p}}\left( {{\text{y}}_{{\text{i}}} } \right)} \right) + (1 - {\text{y}}_{{\text{i}}} ) \times \log \left( {1 - {\text{p}}\left( {{\text{y}}_{{\text{i}}} } \right)} \right)$ Where N is the number of samples (6)	How closely the prediction probability matches the true value is indicated by log loss.
9	Mathew’s correlation coefficient (MCC)	${\text{MCC}} = \frac{{{\text{TP}} \times {\text{TN}} - {\text{FP}} \times {\text{FN}}}}{{\sqrt {({\text{TP}} + {\text{FP}})({\text{TP}} + {\text{FN}})({\text{TN}} + {\text{FP}})({\text{TN}} + {\text{FN}})} }}$ (7)	It measures the difference between the actual value and predicted value.

## Results

The culmination of our comprehensive methodology unfolds in the presentation of results and the ensuing discussions, shedding light on the efficacy of our approach in anemia diagnosis. The robustness of our machine learning models is underscored by their ability to accurately classify anemia types, a critical facet in personalized healthcare. The high-confidence predictions instill confidence in the model’s reliability, particularly in scenarios where precise anemia classification is imperative for tailored treatment strategies.

The presentation of data and subsequent discussions represent the pinnacle of our thorough technique and provide insight into the effectiveness of our approach in diagnosing anemia. The ability of our ML algorithms to reliably distinguish anemia types is a crucial component of individualized healthcare highlights their robustness. The model’s dependability is bolstered by the high confidence predictions, especially in situations when accurate anemia classification is essential for customized treatment plans.

A snapshot of the predictions made by our model is contained in the Table 6. Notably, our model demonstrates the high confidence in cases where the predicted and actual anemia types align, exemplified by instances of correctly identifying IDA and AA. The highest accuracy of 96% is obtained here from LGBM and Stacked models. The accuracy obtained from Logistic regression, Random Forest, Decision tree, KNN, Adaboost, Catboost and Xgboost are 91%, 92%, 90%, 87%, 92%, 94%, and 94% respectively. The tree-based models better than the other baseline models since the data was non-linear in nature.

**Table 6:** Results obtained from each model.

Algorithms	Accuracy	Precision	Recall	F1 score	Hamming loss	Jaccard score	Log loss	Mathews correlation co-efficient
Logistic regression	0.91	0.85	0.95	0.9	0.09	0.81	3.24	0.82
Random forest	0.92	0.87	0.95	0.91	0.08	0.83	2.88	0.84
Decision tree	0.9	0.83	0.95	0.89	0.1	0.8	3.6	0.8
KNN	0.87	0.82	0.88	0.85	0.13	0.73	4.68	0.74
Adaboost	0.92	0.88	0.93	0.9	0.08	0.83	2.88	0.84
Cat boost	0.94	0.89	0.98	0.93	0.06	0.87	2.16	0.88
Light GBM	0.96	0.95	0.95	0.95	0.04	0.91	1.44	0.92
Xg boost	0.94	0.91	0.99	0.93	0.06	0.87	2.16	0.88
Stack	0.96	0.95	0.95	0.95	0.04	0.91	1.44	0.92
ANN	0.95	0.98	0.91	0.94	0.05	0.89	1.73	0.90

**Fig. 9:**
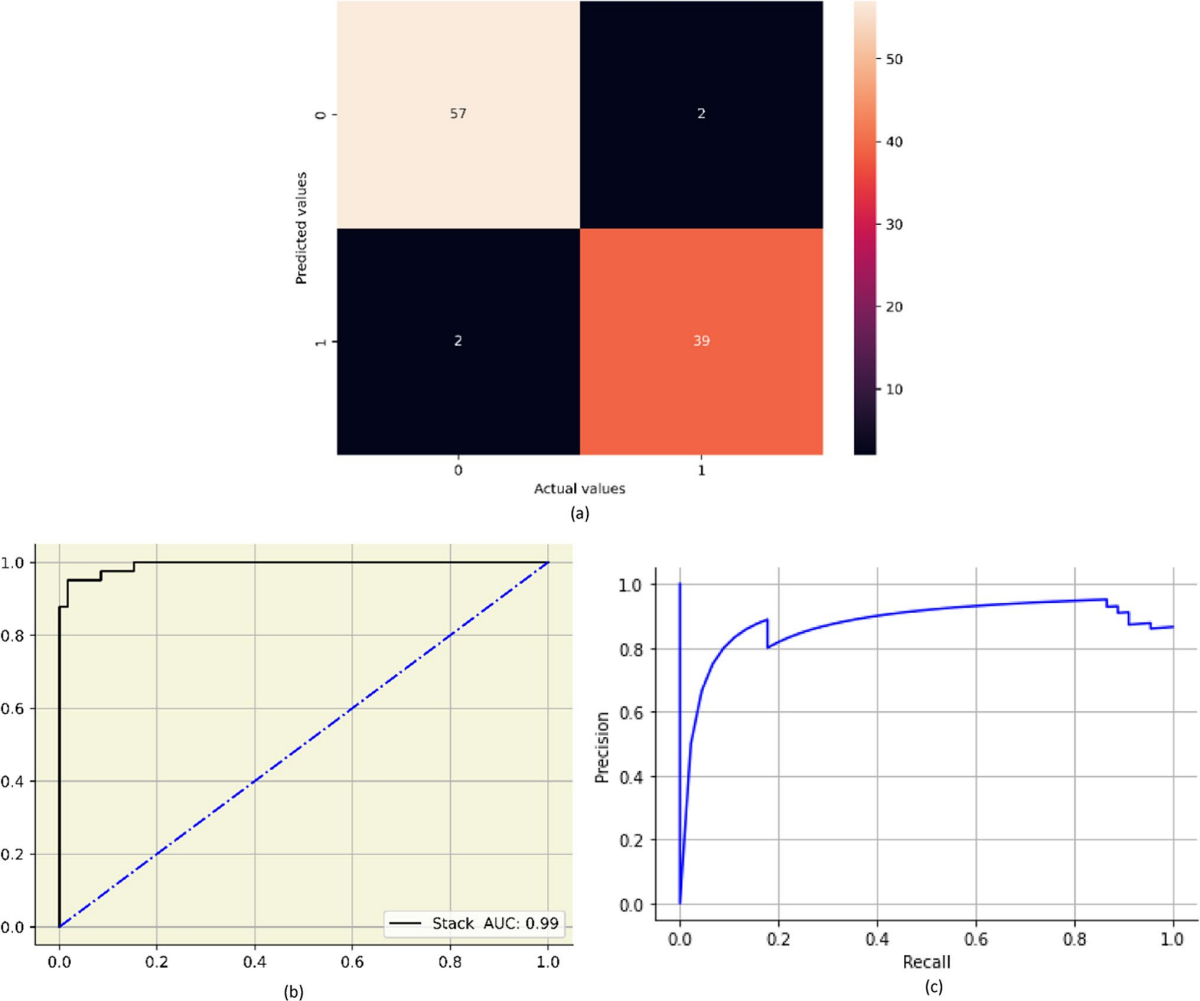
(**a**) Confusion matrix of stack (**b**) AUC curve of stack (**c**) Precision recall curve of stack model.

The confusion matrix of the stacked model is shown in the Fig. 9 (a) and the AUC curve and Precision recall curve of the final stacked model is shown in the Fig. 9 (b) and (c) respectively.

**Fig. 10:**
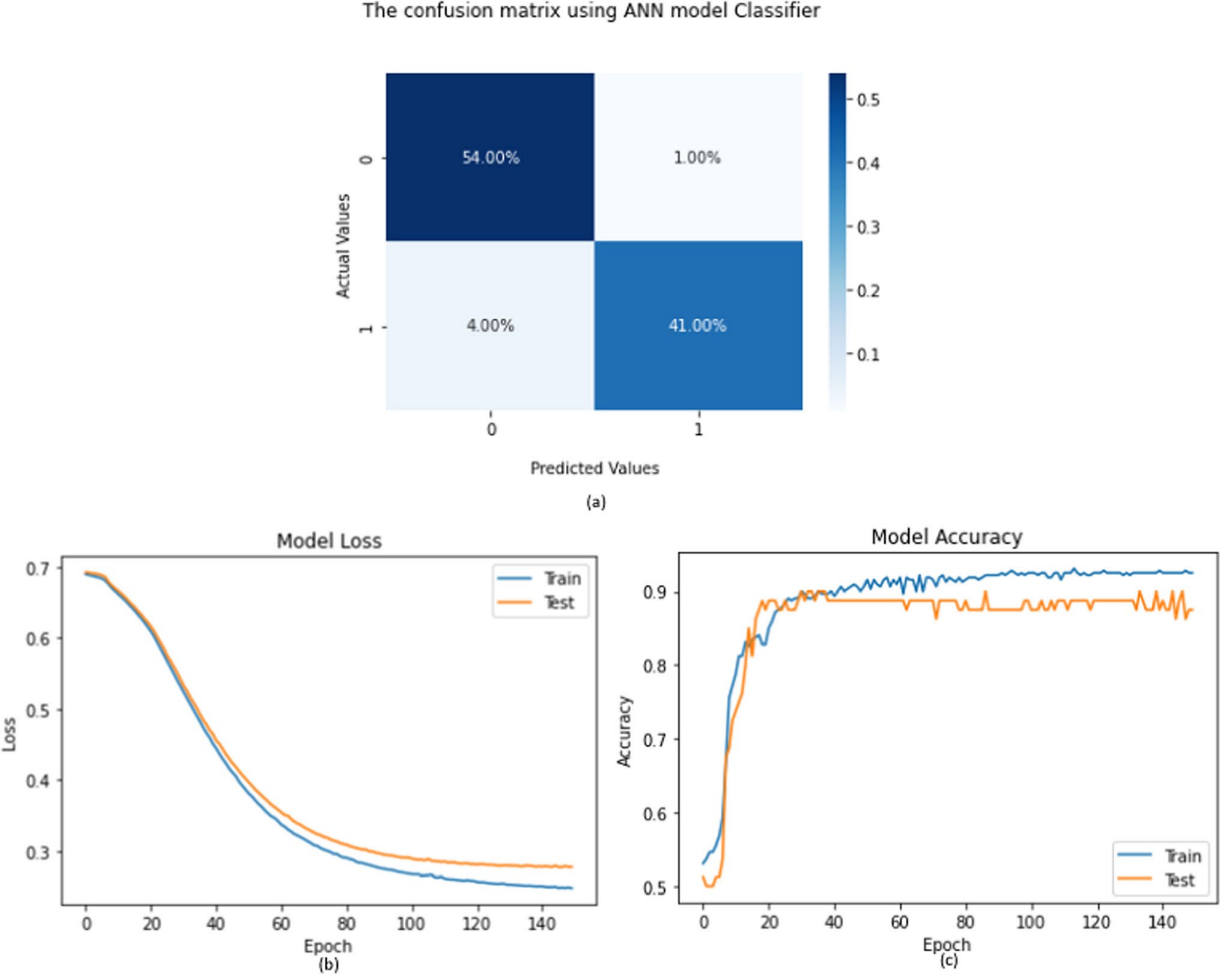
(**a**) Confusion matrix of ANN (**b**) Loss curve of ANN (**c**) Accuracy curve of ANN.

The accuracy obtained from the DL model that is ANN is 95%. The loss curve is shown in the Fig. 10 (b) and the accuracy curve is shown in Fig. 10 (c). The confusion matrix using ANN model classifier can be observed from the Fig. 10(a).

The integration of Explainable AI techniques, including SHAP, LIME, ELi5, Anchor, and Q Lattice, has not only facilitated accurate predictions but has also brought transparency to the decision-making process. Interpretability subsets generated by these techniques offer insights into the features influencing model predictions. For instance, SHAP analysis reveals the pivotal role of hemoglobin levels in determining anemia types, contributing to our understanding of the model’s decision rationale.

As we traverse the landscape of ethical considerations and bias mitigation, the results affirm our commitment to fairness and responsible AI implementation. Stakeholder engagement and collaboration with healthcare professionals have further refined the models, ensuring alignment with clinical standards and augmenting the practical applicability of our research.

In conclusion, our methodology has not only demonstrated promising results in anemia diagnosis but has also contributed to the discourse on responsible and effective AI deployment in healthcare. The convergence of machine learning, Explainable AI, and domain expertise paves the way for a nuanced and transparent approach to medical decision-making.

Decisions about healthcare will be greatly influenced by the diagnosis provided by the classification system. Numerous procedures and operations have been automated and digitized because of technology breakthroughs. Consequently, methods that are precise, comprehensible, and straightforward are prioritized more highly.

In the complex field of medicine, a healthcare professional’s ability to validate the suggested predictions is enhanced by an understandable XAI model. Evaluating the diagnostic model’s performance is crucial before deciding on a course of action. Resilient systems also require feature evaluations that consider a variety of aspects. Five explainers have been used in this research. They are SHAP, LIME, Eli5, Qlattice, and Anchor. Many of the explainers used in the creation of this work did not support DL models. In addition, the ML pipelines outperformed the DL models in terms of results. Therefore, in this study, DL models were not exposed to XAI approaches.

**Fig. 11:**
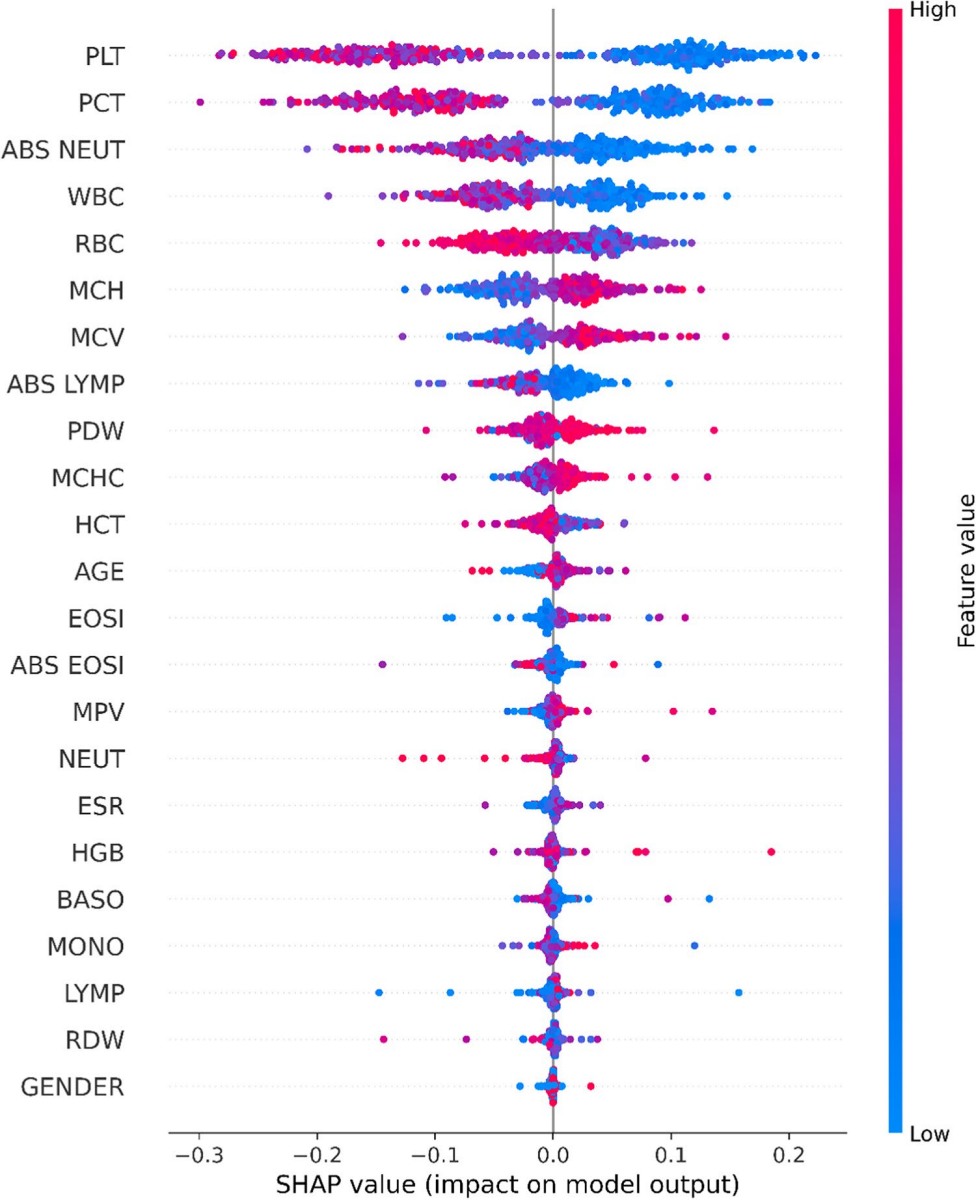
Bee swarm plot using SHAP.

Figure 11 describes the SHAP beeswarm plot for the final customized ensemble model^46^. The two classes are divided by a hyperplane, with Aplastic anemia cases to the right of the hyperplane and Iron deficiency anemia cases to the left of it. Furthermore, higher, and lower values are shown by the colors red and blue respectively. Additionally, the markers are arranged in descending order of significance. The best attribute remains at the top. The plot demonstrates the markers such as PLT, PCT, ABS NEUT, WBC, RBC, MCH, MCV etc. are important. It is also evident from the plot that Aplastic anemia patients have a drop in the platelet (PLT) count whereas in Iron deficiency anemia the platelet count increases. Other attributes such as PCT, ABS NEUT and WBC decreases in aplastic anemia patients and increases in IDA patients. MCV is higher in case of AA and lower in IDA. Other increased markers in Aplastic anemia cases are MCH, MCHC and PDW. PCT, ABS NEUT, WBC, RBC, PDW, and HCT are increased in case of IDA.

**Fig. 12:**
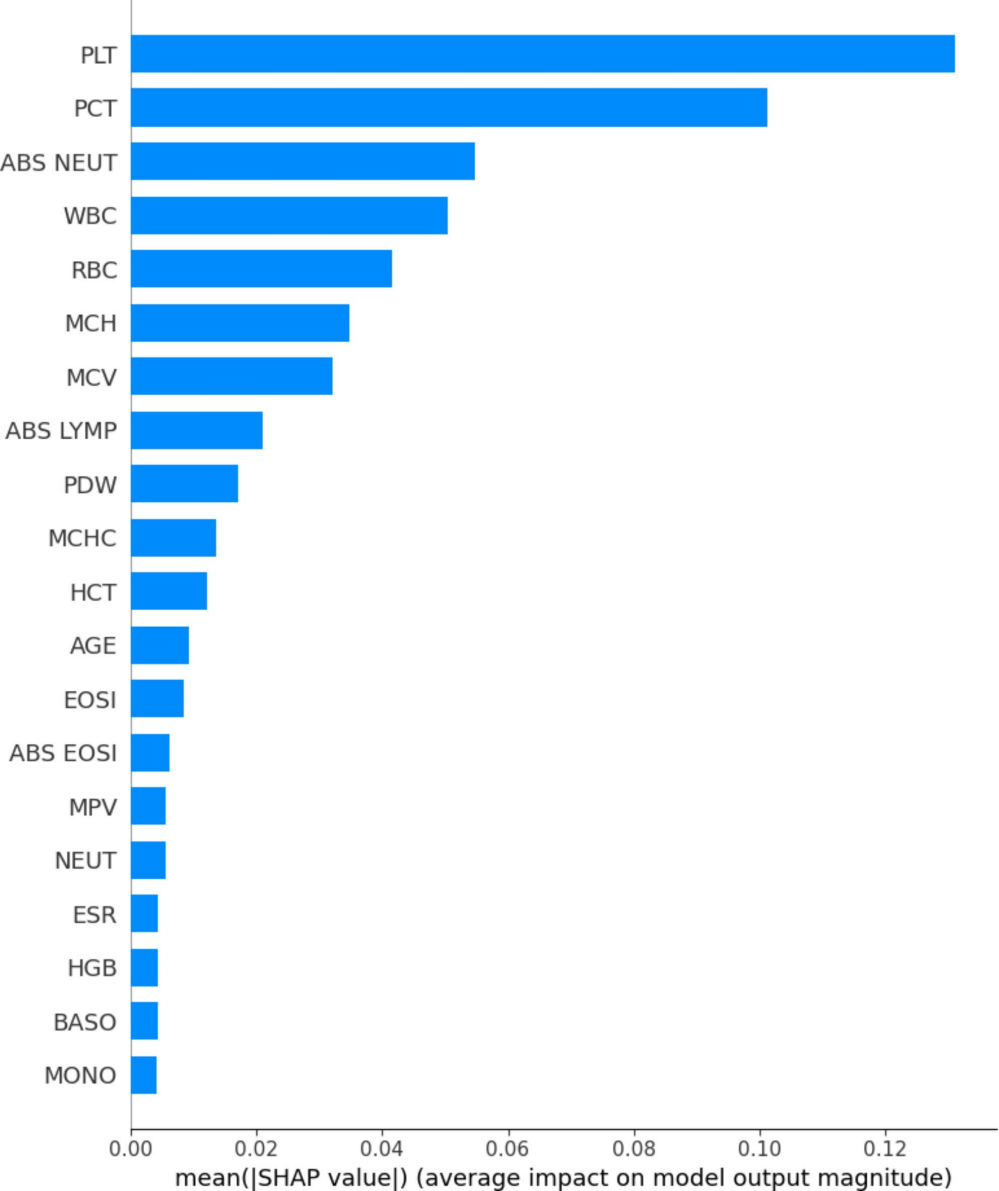
Bar Chart using SHAP.

According to SHAP, PLT, PCT, ABS NEUT, WBC, RBC, MCH, and MCV are essential for differentiating Aplastic Anemia and Iron Deficiency Anemia. In this study, patients with Aplastic Anemia had higher value of MCV. PLT, PCT and RBC is value is higher in Iron deficiency anemia. ABS LYMP, PDW, MCHC, and HCT are other important attributes. Figure 12 displays the mean impact of SHAP values on the output magnitude of the classifier.

**Fig. 13:**
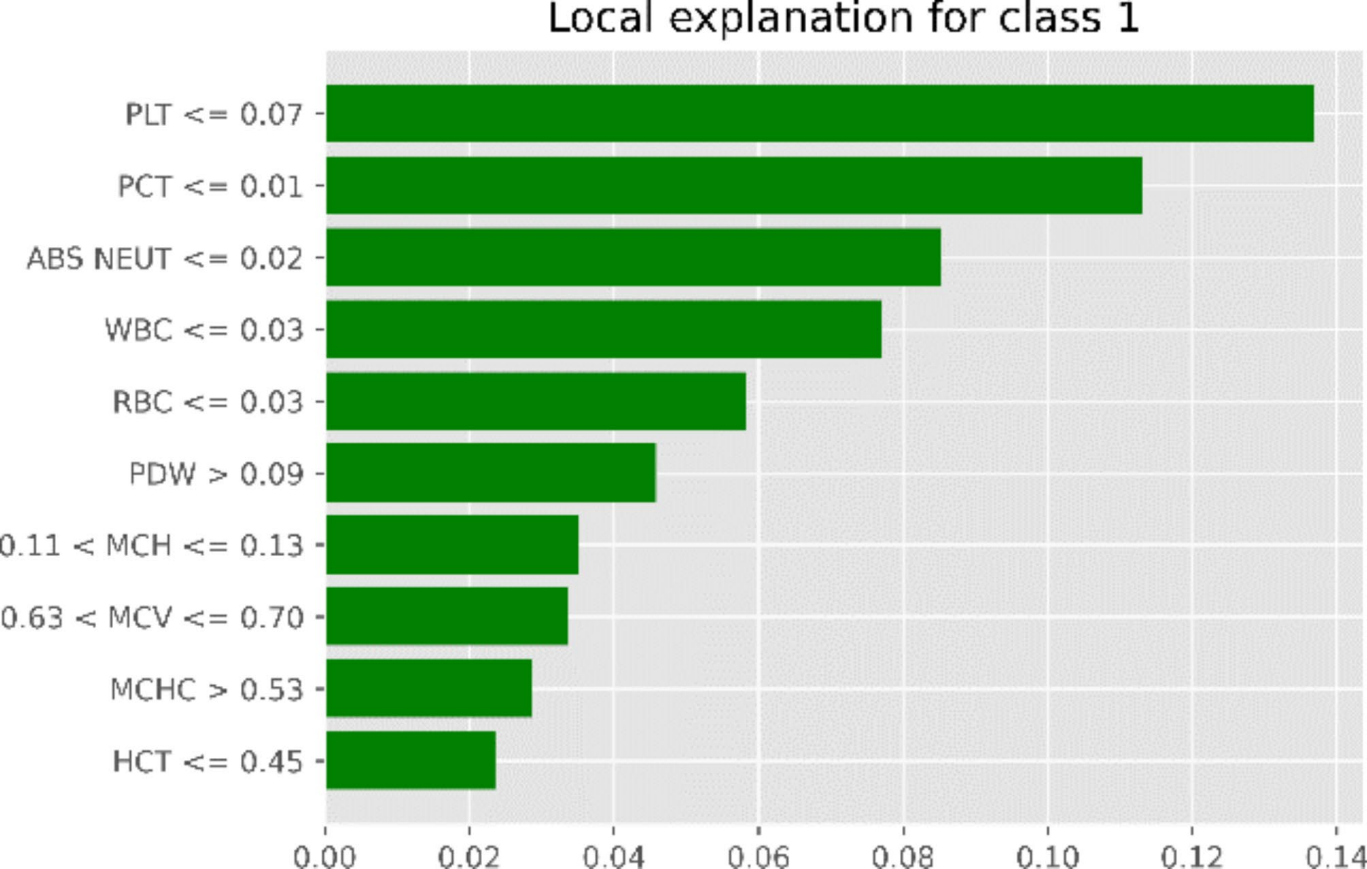
LIME conditions.

LIME is also capable of understanding the ML classifier’s output. After the model has produced its predictions, attributes that require explanation are first selected, and the original data are then altered to make sense of the model’s conclusions. Weights for the new data points need to be assigned based on how close their pertinent occurrences are to one another. The models generate many combinations that are used in training. Lastly, the predictions are explained, and their meaning is interpreted^47^. Aplastic anemia patient’s LIME interpretation is shown in Fig. 13. It is evident that attributes such as PLT, PCT, ABS NEUT and WBC indicates the Aplastic anemia diagnosis. The weights of the attributes are considered when each attribute predict a distinct diagnosis. RBC, MCV, and MCH attributes indicates Iron deficiency anemia diagnosis.

**Fig. 14:**
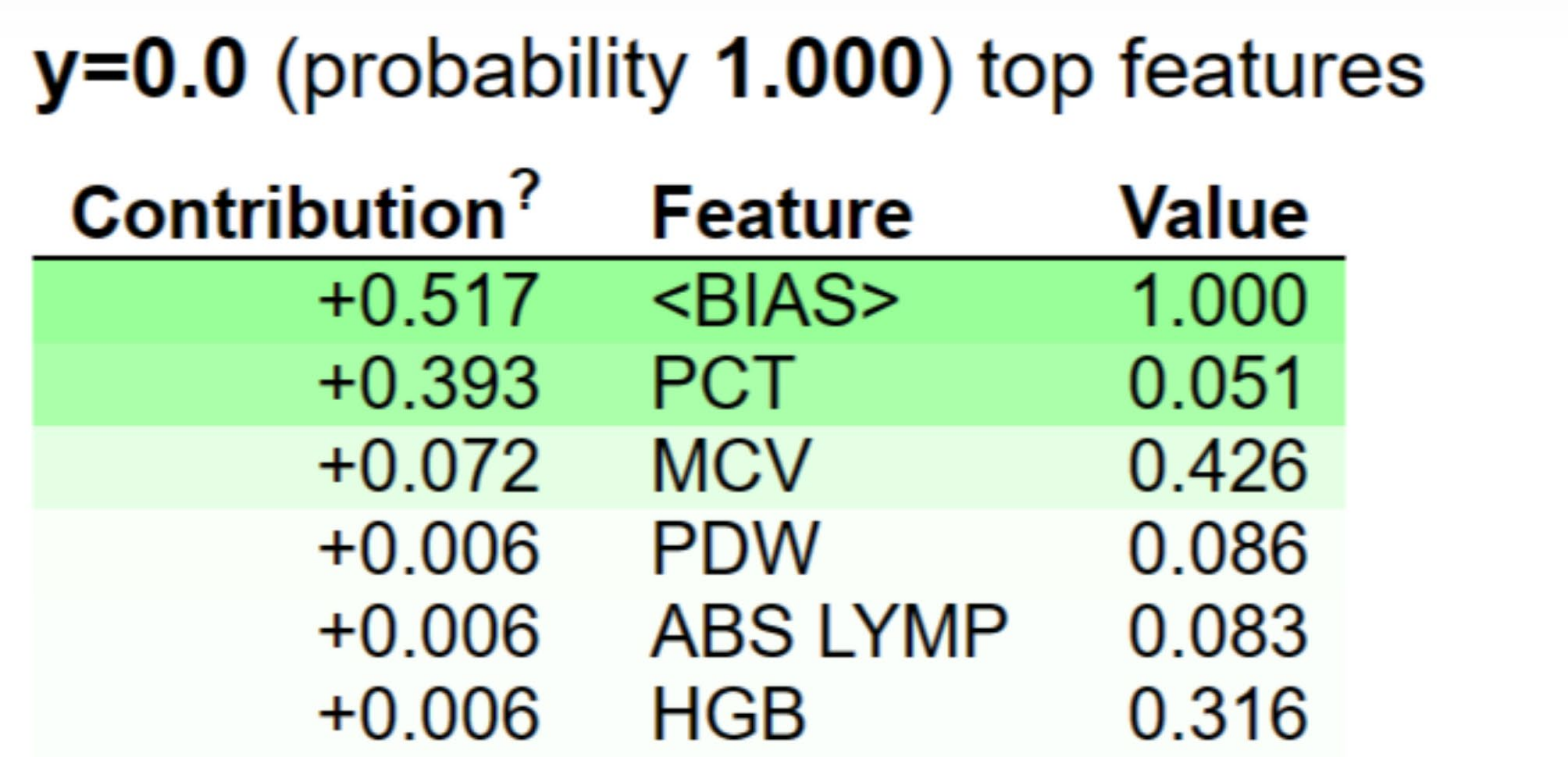
Top features using Eli5.

Eli5 is an another XAI technique for prediction analysis and justification. It uses APIs to visualize and debug predictions. This makes it possible for the researchers to grasp different classifiers when trying to understand predictions^48^. The explainability offered by the Eli5 model is seen in the Fig. 14. The most significant attributes are PCT, MCV, PDW, ABS LYMP, and HGB acoording to Eli5 model. Eli5 takes the bias parameter into account while describing the model.

**Fig. 15:**
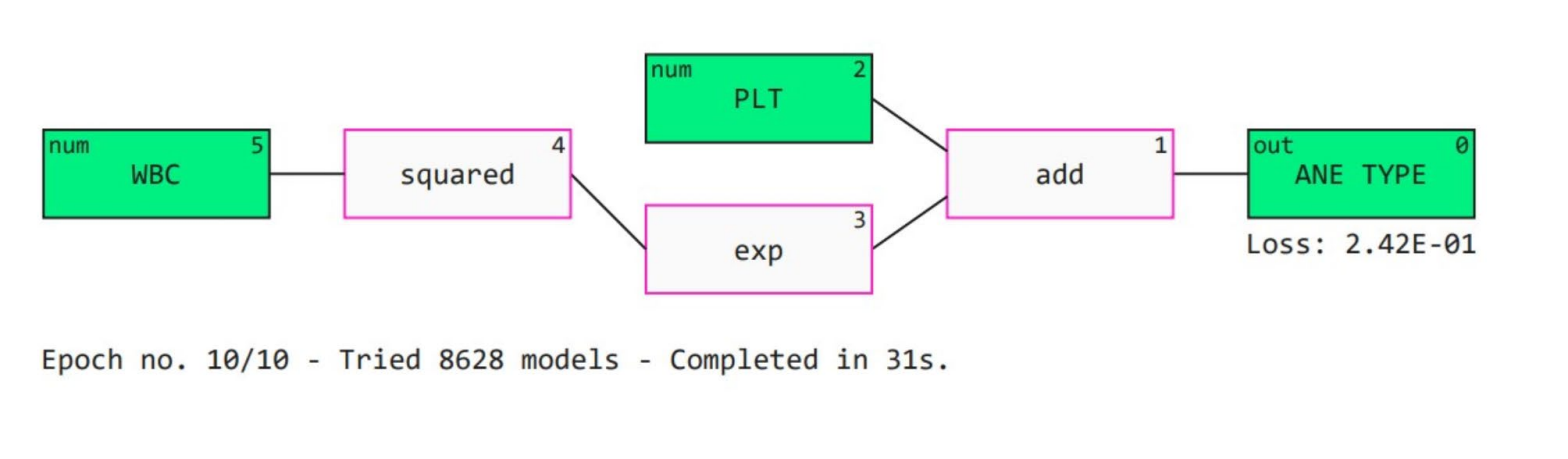
Qlattice output.

In ML, a transparent architecture known as Qlattice is relatively recent. This provides a comprehensive explainability of the “blackbox” idea seen in traditional models. Thousands of possible models are screened by Qlattice before selecting the model that best matches the situation. A few factors, such as input properties and other variables, must initially be configured by the user. The characteristics are referred to as registers in this method. The generated model is referred as a “Qgraph”. There are nodes and edges in the graph. Every node has an activation function given to it, and every edge has a weight. Important details about the properties are produced once the Qgraph is fully trained. In python, Qlattice is installed using the “Feyn” module^49^. A Qgraph is depicted in the Fig. 15. It is evident from the figure that WBC and PLT are considered as the most significant attributes by the model. The “squared”, “exp” and “add” functions are also used by this model to interpret the results.

An additional XAI technique for interpreting ML models is called “Anchor”. Anchors explain the key characteristic using set of “rules” and conditions. Two metrics are used to evaluate each anchor condition: Precision and Coverage. Precision defines the accuracy of the explanations. Coverage determines the quantity of occurrences that employ the same condition for prediction^50^. Table 7 explains anchor conditions. The most effective indicators of Aplastic anemia are PCT, RBC, WBC, MCV, ABS NEUT and PLT. The most effective indicators for identifying Iron Deficiency Anemia are PCT, WBC, MCV, PLT and MCHC.

**Table 7:** Anchor conditions.

Instance	Patient prediction	Anchor condition	Precision	Coverage
1	Iron deficiency anemia	PCT > 0.03 AND MCV < = 0.54	0.99	0.22
2	Iron deficiency anemia	PCT > 0.05 AND MCV < = 0.54	1.00	0.14
3	Iron deficiency anemia	PCT > 0.03 AND MCV < = 0.54	0.99	0.22
4	Iron deficiency anemia	PCT < = 0.01 AND PLT < = 0.06	0.95	0.24
5	Iron deficiency anemia	PCT > 0.03 AND MCHC < = 0.49	0.93	0.19
6	Aplastic anemia	PCT < = 0.01 AND RBC < = 0.03	0.90	0.16
7	Aplastic anemia	WBC < = 0.03 AND PCT < = 0.01	0.97	0.17
8	Aplastic anemia	MCV > 0.63 AND PCT < = 0.01	0.98	0.22
9	Aplastic anemia	ABS NEUT < = 0.02 AND PCT < = 0.01	0.98	0.16
10	Aplastic anemia	PCT < = 0.03 AND PLT < = 0.06	0.94	0.26
11	Aplastic anemia	PCT < = 0.03 AND PLT < = 0.18	0.85	0.49

## Discussion

ML was used in this work to assess a patient’s risk for IDA and AA. There were 500 patients in the dataset. The feature selection techniques used here are Pearson’s Correlation and Mutual Information. Decision tree, Logistic Regression, Random Forest, KNN, Stack 1(DT, RF, LR, KNN), Adaboost, Catboost, LGBM, Xgboost, Stack 2 and Final Stack are the ML models used in this work. ANN, a deep learning algorithm was also used for the model evaluation. Five XAI methodologies were used, and a comparison of the approaches was done to improve the understanding of the results. Using the ML model, anemia prediction is possible as a first step in a Decision support system. The final stack model was picked for prediction as it outperformed all other models. Five XAI models -SHAP, Eli5, LIME, Qlattice and Anchor were employed, and contrasted to better understanding of the results.

By knowing some features of the blood attributes such as HGB, MCV, RBC count etc., the anemic condition of a patient is determined. ML models that asses’ medical data are used to forecast anemic condition. Other important features of the blood test are also considered by these models to improve forecasts.

In recent studies, the XAI models are not much utilized. Mohammed et al. used Naive bayes, LR, Bayesian network and Multilayer perceptron models for the analysis of anemia. They got highest accuracy of 87.3%. No XAI methods are used in this study. Yildiz et al. used ANN, SVM. NB, Ensemble decision trees to classify anemia types. They got an accuracy of 98.4%. They did not incorporate any XAI technique in their study. Kovacevic et al. used KNN as an application of AI in diagnosis of anemia without utilizing any XAI techniques. Prajapathi et al. used Xgboost technique for the classification and analysis of anemia. They got an accuracy of 96.95% and used one XAI model called SHAP in their study. Here in this research, we have used different ML models and Ensemble models along with the DL model ANN. The highest accuracy of 96% is also obtained. Five different XAI methods such as SHAP, LIME, Eli5, Qlattice, and Anchor are used in this study to comprehend the results. PCT, MCV, PDW, ABS LYMP, HGB, WBC are considered as the prominent attributes in both IDA and AA. Table 8 compares the recent works on anemia classification with our work.

**Table 8:** Comparison of recent works.

Sl no	Reference	Classifiers	Accuracy	XAI techniques
1	Mohammed^51^	NB, LR, Bayesian network, MLP	87.3%	No
2	Yildiz^52^	ANN, SVM, NB, ENSEMBLE DECISION TREES.	85.6%	No
3	Kovacevic^53^	KNN	98.4%	No
4	Prajapathi^54^	XGB	96.95%	SHAP only
5	Proposed model	**LR**,** RF**,** DT**,** KNN**,** ADB**,** CTB**,** LGBM**,** XGB**,** ANN**,** Stack**	**91%**,** 92%**,** 90%**,** 87%**,** 92%**,** 94%**,** 96% 94%**,** 95%**,** 96%**	**SHAP**,** LIME**,** Qlattice**,** Eli5**,** Anchor**

Significant values are in [bold].

## Limitations and future scope

The model’s performance is dependent on the availability and quality if the datasets, thus it must be updated and expanded over time. Before being implemented in healthcare facilities, extensive testing, scalability evaluations, and external validations are necessary to ensure resilience across a range of clinical scenarios. Even with XAI, careful interpretation of the model’s predictions is required for interpretability due to the inherent intricacy of anemia prognosis. Patient’s privacy, regulatory compliance, and ethical concerns are crucial and demand ongoing attention during real-world implementation. These factors demonstrate how important it is to apply a thorough and cautious approach in translating our research into beneficial medicinal applications.

Our proposed study uses integrated method to improve the anemia prediction model in the future. We will concentrate on adding sophisticated features, adding to the dataset over time by working with various medical institutes, and combining the newest imaging modalities. Utilizing cutting-edge transfer learning strategies and deep learning algorithms will be essential, particularly when working with big datasets. To create a globally representative program for anemia research, it is also possible to collaborate internationally to merge data from many nations. Our published study is very relevant to the current era where AI is thriving in the healthcare domain^55–57^.

## Conclusion

Anemia patients can have a better prognosis and experience a lower risk of repercussions with early detection and treatment. Therefore, based on the blood test attributes, we employed ML and XAI approaches to predict IDA and AA. Beyond improving transparency and interpretability, XAI serves as a link between decision-makers in the real world and the technical complexities of the ML models. It addresses practical, ethical, and regulatory issues while enabling people to make sense of complicated forecast and promoting a mutually beneficial partnership between human judgement and AI skills.

The dataset used in this study included 500 patients with 23 attributes of the blood test. Two feature selection methods were applied they are Pearson’s Correlation, and Mutual Information. The accuracy of LGBM and stacked model was 96% and highest among all the models used. SHAP, LIME, Eli5, Qlattice and Anchor are the five techniques utilized to analyze the model’s predictions.

PCT, MCV, RDW, ABS LYMP, HGB, WBC, MCH, ABS NEUT, MCHC, HCT, AGE, PLT, PDW were found to be the most significant predictors of IDA and AA. In addition, the effectiveness of establishing classifier dependability was assessed by comparing the proposed approach with other pertinent studies. Medical practitioners can use these models as a decision support system to anticipate Anemia. This technology could be used to forecast anemia in a larger population by implementing real-time anemia screening using an interface.

## Data Availability

The data will be made available by Mr. Dhruva Darshan B S after obtaining the prior permissions from Manipal Academy of Higher Education.

## References

[1] Vieth, J. T. & Lane, D. R. Anemia. *Emerg. Med. Clin*. **32** (3), 613–628. (2014). 10.1016/j.emc.2014.04.007 25060253

[2] World Health Organization. Worldwide prevalence of anaemia 1993–2005: WHO global database on anaemia. (2008).

[3] Milman, N. Anemia—still a major health problem in many parts of the world! *Ann. Hematol.* **90**, 369–377 (2011). 21221586 10.1007/s00277-010-1144-5

[4] Salive, M. E. et al. Anemia and hemoglobin levels in older persons: Relationship with age, gender, and health status. *J. Am. Geriatr. Soc.* **40** (5), 489–496 (1992). 1634703 10.1111/j.1532-5415.1992.tb02017.x

[5] Cappellini, M. D. & Motta, I. Anemia in clinical practice—definition and classification: Does hemoglobin change with aging?. *Seminars Hematol*. **52** (4), 261–269 (2015). 10.1053/j.seminhematol.2015.07.006 26404438

[6] Bessman, J. D., Gilmer, P. R. Jr & Gardner, F. H. Improved classification of anemias by MCV and RDW. *Am. J. Clin. Pathol.* **80** (3), 322–326 (1983). 6881096 10.1093/ajcp/80.3.322

[7] Hess, S. Y. et al. Accelerating action to reduce anemia: Review of causes and risk factors and related data needs. *Ann. N. Y. Acad. Sci*. **1523** (1), 11–23 (2023). 36987993 10.1111/nyas.14985 PMC10918744

[8] Gjørup, T., Bugge, P. M., Hendriksen, C., & Jensen, A. M. A critical evaluation of the clinical diagnosis of anemia. *Am. J. Epidemiol*. **124** (4), 657–665 (1986). 3752058 10.1093/oxfordjournals.aje.a114438

[9] Meena, G., Mohbey, K. K., Acharya, M. & Lokesh, K. Original research article an improved convolutional neural network-based model for detecting brain tumors from augmented MRI images. *J. Auton. Intell*. **6** (1) (2023).

[10] Alanazi, A. Using machine learning for healthcare challenges and opportunities. *Inf. Med. Unlocked*. **30**, 100924 (2022).

[11] Gerlings, J., Jensen, M. S. & Shollo, A. Explainable AI, but explainable to whom? An exploratory case study of xAI in healthcare. In *Handbook of Artificial Intelligence in Healthcare* Vol. 2 169–198 Practicalities and Prospects. (2022).

[12] Arrieta, A. B. et al. Explainable Artificial Intelligence (XAI): Concepts, taxonomies, opportunities and challenges toward responsible AI. *Inform. Fusion*. **58**, 82–115 (2020).

[13] Kilicarslan, S., Celik, M. & Sahin, Ş. Hybrid models based on genetic algorithm and deep learning algorithms for nutritional anemia disease classification. *Biomed. Signal Process. Control*. **63**, 102231 (2021).

[14] Zhang, A. et al. Prediction of anemia using facial images and deep learning technology in the emergency department. *Front. Public. Health*. **10**, 964385 (2022). 36438300 10.3389/fpubh.2022.964385 PMC9682145

[15] Appiahene, P. et al. Application of ensemble models approach in anemia detection using images of the palpable palm. *Med. Novel Technol. Devices*. **20**, 100269 (2023).

[16] Rahman, M. M. et al. Anemia disease prediction using machine learning techniques and performance analysis. In *2024 11th International Conference on Computing for Sustainable Global Development (INDIACom)* 1276–1282 (IEEE, 2024).

[17] Aliyu, H. A., Razak, M. A. A., Sudirman, R. & Ramli, N. A deep learning AlexNet model for classification of red blood cells in sickle cell anemia. *Int. J. Artif. Intell.* **9** (2), 221–228 (2020).

[18] Kinyoki, D., Osgood-Zimmerman, A. E., Bhattacharjee, N. V., Kassebaum, N. J. & Hay, S. I. Anemia prevalence in women of reproductive age in low-and middle-income countries between 2000 and 2018. *Nat. Med.* **27** (10), 1761–1782 (2021). 34642490 10.1038/s41591-021-01498-0 PMC8516651

[19] Dejene, B. E., Abuhay, T. M. & Bogale, D. S. Predicting the level of anemia among Ethiopian pregnant women using homogeneous ensemble machine learning algorithm. *BMC Med. Inf. Decis. Mak.* **22** (1), 247 (2022). 10.1186/s12911-022-01992-6 PMC9494842 36138398

[20] Appiahene, P., Asare, J. W., Donkoh, E. T., Dimauro, G. & Maglietta, R. Detection of iron deficiency anemia by medical images: A comparative study of machine learning algorithms. *BioData Min.* **16** (1), 2 (2023). 36694237 10.1186/s13040-023-00319-z PMC9875467

[21] Shweta, N. & Pande, S. D. Prediction of anemia using various ensemble learning and boosting techniques. *EAI Endorsed Trans. Pervasive Health Technol*. **9** (1) (2023).

[22] Zemariam, A. B. et al. Employing supervised machine learning algorithms for classification and prediction of anemia among youth girls in Ethiopia. *Sci. Rep.* **14** (1), 9080 (2024). 38643324 10.1038/s41598-024-60027-4 PMC11032364

[23] Rahman, M. et al. Anemia disease prediction using machine learning techniques and performance analysis. In *2024 11th International Conference on Computing for Sustainable Global Development (INDIACom)* 1276–1282 (IEEE, 2024).

[24] Qasrawi, R. et al. Identification and prediction of association patterns between nutrient intake and anemia using machine learning techniques: Results from a cross-sectional study with university female students from Palestine. *Eur. J. Nutr*. 1–15. (2024). 10.1007/s00394-024-03360-8 PMC11329411 38512358

[25] Pullakhandam, S. & McRoy, S. Classification and explanation of iron deficiency anemia from complete blood count data using machine learning. *BioMedInformatics* **4** (1), 661–672 (2024).

[26] Asare, J. W., Brown-Acquaye, W. L., Ujakpa, M. M., Freeman, E. & Appiahene, P. Application of machine learning approach for iron deficiency anaemia detection in children using conjunctiva images. *Inf. Med. Unlocked*. **45**, 101451 (2024).

[27] Saputra, D. C. E., Sunat, K. & Ratnaningsih, T. A new artificial intelligence approach using extreme learning machine as the potentially effective model to predict and analyze the diagnosis of anemia. *Healthcare*. **11** (5), 697 (2023). 36900702 10.3390/healthcare11050697 PMC10000789

[28] Kebede Kassaw, A., Yimer, A., Abey, W., Molla, T. L. & Zemariam, A. B. The application of machine learning approaches to determine the predictors of anemia among under five children in Ethiopia. *Sci. Rep.* **13** (1), 22919 (2023). 38129535 10.1038/s41598-023-50128-x PMC10739802

[29] Shehab, E. & Khawaga, A. Anemia diagnosis and prediction based on machine learning. *Kafrelsheikh J. Inform. Sci.* **4** (2), 1–9 (2023).

[30] Zahirzada, A., Zaheer, N. & Shahpoor, M. A. Machine learning algorithms to predict anemia in children under the age of five years in Afghanistan: A case of Kunduz Province. *J. Surv. Fisheries Sci.* **10** (4S), 752–762 (2023).

[31] Milanes-Baños, N. A. Step-by-step one-way ANOVA analysis with the Jamovi Program. *Mexican J. Med. Res. ICSA* (2024).

[32] Tanious, R. & Manolov, R. Violin plots as visual tools in the meta-analysis of single-case experimental designs. *Methodology* **18** (3), 221–238 (2022).

[33] Lee, J. Y., Kerns, S. & Wilmer, J. Bar graphs of mean values produce inflated and variable estimates of effect size. *J. Vis.* **22** (14), 4432–4432 (2022).

[34] Dietterich, T. G. Machine learning for sequential data: A review. In Structural, Syntactic, and Statistical Pattern Recognition: Joint IAPR International Workshops SSPR 2002 and SPR 2002 Windsor, Ontario, Canada, August 6–9, 2002 Proceedings 15–30. (Springer, Berlin, 2002).

[35] Cohen, I. et al. Pearson correlation coefficient. In *Noise reduction in speech processing*, 1–4. (2009).

[36] Estévez, P. A., Tesmer, M., Perez, C. A. & Zurada, J. M. Normalized mutual information feature selection. *IEEE Trans. Neural Netw*. **20** (2), 189–201 (2009). 19150792 10.1109/TNN.2008.2005601

[37] Ali, P. J. M., Faraj, R. H., Koya, E., Ali, P. J. M. & Faraj, R. H. Data normalization and standardization: A technical report. *Mach. Learn. Tech. Rep.* **1** (1), 1–6 (2014).

[38] Rattan, V., Mittal, R., Singh, J. & Malik, V. Analyzing the application of SMOTE on machine learning classifiers. In *2021 International Conference on Emerging Smart Computing and Informatics (ESCI)* 692–695 (IEEE, 2021).

[39] Probst, P., Boulesteix, A. L. & Bischl, B. Tunability: Importance of hyperparameters of machine learning algorithms. *J. Mach. Learn. Res.* **20** (53), 1–32 (2019).

[40] Bonaccorso, G. *Machine Learning Algorithms: Popular Algorithms for data Science and Machine Learning* (Packt Publishing Ltd., 2018).

[41] Pavlyshenko, B. Using stacking approaches for machine learning models. In *2018 IEEE second international conference on data stream mining & processing (DSMP)* 255–258 (IEEE, 2018).

[42] Shahhosseini, M., Hu, G. & Pham, H. Optimizing ensemble weights and hyperparameters of machine learning models for regression problems. *Mach. Learn. Appl.* **7**, 100251 (2022).

[43] Sarker, I. H. Deep learning: A comprehensive overview on techniques, taxonomy, applications and research directions. *SN Comput. Sci.* **2** (6), 420 (2021). 34426802 10.1007/s42979-021-00815-1 PMC8372231

[44] Chadaga, K. et al. Explainable artificial intelligence approaches for COVID-19 prognosis prediction using clinical markers. *Sci. Rep.* **14** (1), 1783 (2024). 38245638 10.1038/s41598-024-52428-2 PMC10799946

[45] Erickson, B. J. & Kitamura, F. Magician’s corner: 9. Performance metrics for machine learning models. *Radiology: Artif. Intell*. **3**(3), e200126. (2021). 10.1148/ryai.2021200126 PMC8204137 34136815

[46] Meng, Y., Yang, N., Qian, Z. & Zhang, G. What makes an online review more helpful: An interpretation framework using XGBoost and SHAP values. *J. Theoretical Appl. Electron. Commer. Res*. **16** (3), 466–490 (2020).

[47] Garreau, D. & Luxburg, U. Explaining the explainer: A first theoretical analysis of LIME. In *International conference on artificial intelligence and statistics* 1287–1296 (PMLR, 2020).

[48] Fan, A. et al. ELI5: Long form question answering. arXiv preprint arXiv:1907.09190. (2019).

[49] Wenninger, S., Kaymakci, C. & Wiethe, C. Explainable long-term building energy consumption prediction using QLattice. *Appl. Energy*. **308**, 118300 (2022).

[50] Haag, F., Stingl, C., Zerfass, K., Hopf, K. & Staake, T. Overcoming anchoring bias: The potential of AI and XAI-based decision support. arXiv preprint arXiv:2405.04972. (2024).

[51] Mohammed, M. S., Ahmad, A. A. & Murat, S. A. R. I. Analysis of anemia using data mining techniques with risk factors specification. In *2020 International Conference for Emerging Technology (INCET)* 1–5 (IEEE, 2020).

[52] Yıldız, T. K., Yurtay, N. & Öneç, B. Classifying anemia types using artificial learning methods. *Eng. Sci. Technol. Int. J.* **24** (1), 50–70 (2021).

[53] Kovačević, A. et al. Application of artificial intelligence in diagnosis and classification of anemia. In *2022 11th Mediterranean Conference on Embedded Computing (MECO)* 1–4. (IEEE, 2022).

[54] Prajapati, J., Uduthalapally, V., Das, D., Mahapatra, R. & Wasnik, P. N. XAIA: An Explainable AI approach for classification and analysis of blood anemia. In *2023 OITS International Conference on Information Technology (OCIT)* 88–93. (IEEE, 2023).

[55] Meena, G., Mohbey, K. K. & Kumar, S. Monkeypox recognition and prediction from visuals using deep transfer learning-based neural networks. *Multimed.Tools Appl.* **83**, 71695–71719. 10.1007/s11042-024-18437-z (2024).

[56] Meena, G. & Mohbey, K. K. Sentiment analysis on images using different transfer learning models. *Procedia Comput. Sci.* **218**, 1640–1649 (2023).

[57] Meena, G., Indian, A., Mohbey, K. K. & Jangid, K. Point of interest recommendation system using sentiment analysis. *J. Inform. Sci. Theory Pract.* **12** (2), 64–78. 10.1633/JISTaP.2024.12.2.5 (2024).

